# Benchmarking the Cost per Person of Mass Treatment for Selected Neglected Tropical Diseases: An Approach Based on Literature Review and Meta-regression with Web-Based Software Application

**DOI:** 10.1371/journal.pntd.0005037

**Published:** 2016-12-05

**Authors:** Christopher Fitzpatrick, Fiona M. Fleming, Matthew Madin-Warburton, Timm Schneider, Filip Meheus, Kingsley Asiedu, Anthony W. Solomon, Antonio Montresor, Gautam Biswas

**Affiliations:** 1 Department of Control of Neglected Tropical Diseases, World Health Organization, Geneva, Switzerland; 2 Imperial College London, London, United Kingdom; 3 University of York, York, United Kingdom; 4 Universität Düsseldorf, Düsseldorf, Germany; 5 Health Economics Unit, University of Cape Town, Cape Town, South Africa; University of Oklahoma Health Sciences Center, UNITED STATES

## Abstract

**Background:**

Advocacy around mass treatment for the elimination of selected Neglected Tropical Diseases (NTDs) has typically put the cost per person treated at less than US$ 0.50. Whilst useful for advocacy, the focus on a single number misrepresents the complexity of delivering “free” donated medicines to about a billion people across the world. We perform a literature review and meta-regression of the cost per person per round of mass treatment against NTDs. We develop a web-based software application (https://healthy.shinyapps.io/benchmark/) to calculate setting-specific unit costs against which programme budgets and expenditures or results-based pay-outs can be benchmarked.

**Methods:**

We reviewed costing studies of mass treatment for the control, elimination or eradication of lymphatic filariasis, schistosomiasis, soil-transmitted helminthiasis, onchocerciasis, trachoma and yaws. These are the main 6 NTDs for which mass treatment is recommended. We extracted financial and economic unit costs, adjusted to a standard definition and base year. We regressed unit costs on the number of people treated and other explanatory variables. Regression results were used to “predict” country-specific unit cost benchmarks.

**Results:**

We reviewed 56 costing studies and included in the meta-regression 34 studies from 23 countries and 91 sites. Unit costs were found to be very sensitive to economies of scale, and the decision of whether or not to use local volunteers. Financial unit costs are expected to be less than 2015 US$ 0.50 in most countries for programmes that treat 100 thousand people or more. However, for smaller programmes, including those in the “last mile”, or those that cannot rely on local volunteers, both economic and financial unit costs are expected to be higher.

**Discussion:**

The available evidence confirms that mass treatment offers a low cost public health intervention on the path towards universal health coverage. However, more costing studies focussed on elimination are needed. Unit cost benchmarks can help in monitoring value for money in programme plans, budgets and accounts, or in setting a reasonable pay-out for results-based financing mechanisms.

## Author Summary

Advocacy around mass treatment for the elimination of selected Neglected Tropical Diseases (NTDs) has typically put the cost per person treated at less than US$ 0.50. Whilst useful for advocacy, the focus on a single number misrepresents the complexity of delivering “free” donated medicines to about a billion people across the world. Given the increasing focus of the NTD community on value for money and, in the context of universal health coverage, of the global health community on outreach beyond health facilities, there was a need for greater nuance. We performed the most comprehensive literature review and first regression analysis of differences between settings in the cost per person treated against six NTDs (excluding the cost of individual medicines). We considered more than ten possible drivers of cost. We found, for example, that the unit cost of treatment depends very much on the number of people treated (economies of scale). We then developed a web-based software application (https://healthy.shinyapps.io/benchmark/) that can be used to predict setting-specific unit costs against which programme budgets and expenditures or results-based pay-outs can be benchmarked.

## Introduction

Since the year 2006, more than 7 billion treatments against Neglected Tropical Diseases (NTDs) have been delivered to people in need. More than 850 million people were treated in 2014 alone for lymphatic filariasis, schistosomiasis, soil-transmitted helminthiasis, onchocerciasis, and trachoma. Up to 1.4 billion people are targeted for coverage, requiring an investment of an estimated US$ 2.8 billion in the period 2015–2020.[1]

The six abovementioned NTDs are caused by different pathogens (including bacteria and helminths). However, only four drugs (albendazole, azithromycin, ivermectin or diethylcarbamazine and praziquantel) are needed to treat them and the strategy for delivering those medicines to reduce morbidity and prevent transmission is similar.[1] Mass treatment is known formally as Preventive Chemotherapy (PC) or Mass Drug Administration (MDA) in the case of the five abovementioned NTDs, and Total Community Treatment (TCT) in the case of yaws. Mass treatment involves the single-dose administration of (largely donated) medicines to entire populations at risk, without the need for diagnosis.

In advocating for mass treatment against the five PC diseases, the NTD community has typically cited values of US$0.10 to US$0.50 as the delivery cost per person per year. [1] These values exclude the cost of donated medicines. Whilst useful for advocacy, the focus on single numbers risks misrepresenting the complexity of delivering “free” medicines to about a billion people across the world.

In this study we conducted a literature review of existing studies and extracted and standardized estimates of the unit cost of delivering mass treatment (excluding the cost of the individual medicines themselves). We considered the six non-zoonotic NTDs for which mass treatment is recommended by the World Health Organization (WHO). Mass treatment is also recommended for foodborne trematodiases, however, scale up for these diseases is more recent with no current data on the cost of delivery.

We developed a regression model of unit costs to better understand the drivers of variation between the studies. The aim of the study was to use this model to “predict” what unit cost one might expect in different settings, along the lines of what has been done by WHO-CHOICE for estimating unit costs of general health services [2]. We called these predictions benchmarks. Benchmarks are setting-specific unit costs against which programme budgets and expenditures might be compared or benchmarked. The regression results were then used to create a web-based software application for planners, funders and researchers. The benchmarks might also inform the design of payment-by-results type financing mechanisms whereby funders agree to pay for outputs (e.g. the number of people treated) rather than inputs (e.g. personnel and equipment).

## Methods

This study was undertaken in four steps: 1. Literature search and review; 2. Data extraction; 3. Meta-regression; 4. Benchmarking.

### Literature Search and Review

In June 2015, we conducted a search of the available literature on the cost of PC or MDA for the five PC diseases and TCT for yaws. The search was conducted in PubMed, in English; the terms are provided in Supplemental Information (S1 Table). In order to maintain comparability in inputs, the search was limited to papers published since 1990. The initial literature search identified 182 studies.

Titles and abstracts were assessed for inclusion. Criteria for inclusion were that: 1) that the population targeted be either all children or all adults (not, for example, just pregnant women); 2) the intervention be mass treatment, rather than individual (diagnosis and) treatment; 3) that the reported outcome (cost) be based at least in part on primary data (some estimation was allowed) and that sufficient detail be provided to ascertain whether these costs were financial or economic and what portion of the costs could be attributed to medicines.

On this basis, 136 studies were excluded. We reviewed the references of the remaining 47 studies and identified another 7 studies. A list of 54 references was shared with disease-specific focal points within WHO and one further study was proposed from the grey literature.[3] Primary data collected by one of the authors (FMF) was also considered for inclusion; these data are publicly available, as described under *Data Extraction*. A total of 56 full texts were assessed for inclusion in the meta-regression.

Upon reviewing the full texts, we excluded a total of 22 studies, listed in Supplemental Information (S2 Table). Of those, 19 nineteen studies were based on the same cost data as an earlier study or another study reporting more detail. One study did not report the number of people treated. One study provided regional costs with no breakdown by country and one study provided costs for chemotherapy of detected cases only, not mass treatment.

This resulted in a final set of 34 studies being selected for inclusion in the meta-regression. These are listed in Supplemental Information (S2 Table). In December 2015, searches undertaken in English, French and Spanish using Google Scholar produced no additional studies.

### Data Extraction

Unit costs were defined as the cost per person per round, not per disease. We extracted unit costs or divided total costs by the number of people treated in a given year (across all rounds). We removed the medicines component (whether purchased or donated) and converted to base year prices (2015 US$) using the GDP deflator.[4] Definitions of what constituted financial or economic costs varied across the studies. We applied a standard classification according to Table 1. One of the notable differences between financial and economic costs relates to the inclusion of Ministry of Health buildings and staff time. While many studies mentioned patient time and the use of local (village or school) volunteers, few reported their (economic) costs and they were therefore excluded from the analysis. We nonetheless recorded the use of volunteers as a dummy variable for use in the meta-regression.

**Table 1:** Classification of financial and economic unit costs (excluding medicines^1^)

Cost	Financial	Economic
Drug delivery (i.e. shipment)	Yes	Yes
Fuel and maintenance	Yes	Yes
Office and other supplies	Yes	Yes
Office utilities	Yes	Yes
Planning and mapping	Yes	Yes
Project staff salaries	Yes	Yes
Per diems	Yes	Yes
Training	Yes	Yes
Vehicles (rented)	Yes	Yes
Vehicles (new)	Yes (annualized)	Yes (annualized)
Vehicles (existing)	No	Yes (annualized)
Ministry of Health buildings	No	Yes (annualized)
Ministry of Health staff time	No	Yes
Volunteer time	No	No^2^
Treated person’s time or other costs	No	No^3^

^1^ Excludes medicines used for mass treatment as well as for treatment of adverse events, if any, which were limited to early mass treatment with diethylcarbamazine (DEC).

^2^ Estimates of the economic cost of volunteer time were removed from the unit costs of the few studies that did report them.

^3^ Estimates of the economic cost of the treated person’s time or other direct or indirect costs associated with treatment or adverse reactions were removed from the unit costs of the few studies that took a societal perspective.

We extracted the number of people treated, the percentage of the target population that was treated, and other variables described in detail under *Meta-regression*. The coverage percentage was not always reported, nor the target population. For school-based programmes, we took the primary school net enrolment rate as a proxy for coverage. In the other cases, we estimated coverage using national data as reported to WHO.[5]

For study sites at the subnational level, we identified the geographical coordinates (administrative centres in the case of regions or districts) and nearest major cities (>100 000 inhabitants) and calculated the distance between them by road (in kilometres) using Google maps. We also recorded the travel time needed (in minutes by car), though this variable was later discarded as it performed no better than distance in predicting unit costs. In the absence of roads (e.g. in the islands of Papua New Guinea and Vanuatu) we used the flying distance to the nearest city.

Data were collated in a spreadsheet using Microsoft Excel. Data were then imported into R for data analysis and visualization.[6] All data are publicly available at https://healthy.shinyapps.io/benchmark/, through the web-based application software described below.

### Meta-regression

We employed meta-regression to examine whether differences in the average cost per person reported by included studies could be explained by moderator variables related to study methodologies or to the settings in which they were conducted.

Regression analysis was performed with the *plm* package for panel data.

We used the study reference as the cross-sectional unit, which allows us to account for the possible clustering of effects due to methodological differences between studies. We used the year, site and comparator as the longitudinal unit. By comparator we refer to the fact that in any given year and site, a study may report and compare multiple costs: economic versus financial costs, integrated versus standalone costs, school-age children versus total population, or one round versus two rounds of delivery.

Models were fit with both random and fixed (within) effects. However, for the purposes of benchmarking (outside of the sample), it would be problematic to select an appropriate fixed effect. We therefore focus in what follows on the random effects model. The results of the fixed effects model are provided in the Supplemental Information (S4 Table).

Model 1 is a random effects model on the full set of observations;

$${log(ucb}_{it})=\alpha +{eco}_{it}\beta_{1}+{vol}_{it}\beta_{2}+{log(int}_{it})\beta_{3}+log({rds}_{it})\beta_{4}+{yrs}_{it}\beta_{5}+{cov}_{it}\beta_{6}+{sch}_{it}\beta_{7}+\log\left({pop}_{it}\right)\beta_{8}+\log\left({den}_{it}\right)\beta_{9}+{nat}_{it}\beta_{10}+\log\left({gdp}_{it}\right)\beta_{11}+u_{it}+\varepsilon_{it}$$ (1)

Model 2 is a random effects model on the subset of observations that are subnational (regional or district) sites, which allows for the inclusion of the distance variable *dis*;

$$log({ucb}_{it})=\alpha +{eco}_{it}\beta_{1}+{vol}_{it}\beta_{2}+log({int}_{it})\beta_{3}+log({rds}_{it})\beta_{4}+{yrs}_{it}\beta_{5}+{cov}_{it}\beta_{6}+{sch}_{it}\beta_{7}+\log\left({pop}_{it}\right)\beta_{8}+\log\left({den}_{it}\right)\beta_{9}+{nat}_{it}\beta_{10}+\log\left({gdp}_{it}\right)\beta_{11}+sqrt({dis}_{it})\beta_{12}+u_{it}+\varepsilon_{it}$$ (2)

where

*ucb* is the unit cost (per person treated per round) in 2015 US$, excluding medicines and volunteer time; we consider with and without Purchasing Power Parity (PPP) conversion;

*i* = 1,…,*I* studies;

*t* = 1,…,*T* year-site-comparators;

*α* is an invariant intercept;

*eco* is a dummy indicating whether the unit cost is economic (1) or financial (0);

*vol* is the volunteer dummy for use of local volunteers, the cost of which is not included in *ucb*;

*int* is an variable with the number of diseases for which treatment is delivered within a given round;

*rds* is the average number of rounds per year;

*yrs* is the number of years during which the programme has been implemented;

*cov* is the percent coverage, or the number of people treated divided by the number of people targeted;

*sch* is a dummy indicating school-based treatment of school children only;

*pop* is the number of people treated per round;

*den* is the population density, defined as the total population divided by the land area, or people per km^2^;

*nat* is a dummy variable indicating a national programme rather than a subnational site;

*gdp* is Gross Domestic Product (GDP) per capita in 2015 US$; with and without PPP conversion;[7]

*dis* is the distance in km from the study site to the nearest city of >100 000 inhabitants;

*u*_*it*_ is the between-study error; and

*ε*_*it*_ is the within-study error.

We consider also the possibility of study- and country-specific dummy variables, as well as interactions between variables, as described in the *Results*.

### Benchmarking

We used the resulting regression model coefficients to estimate or predict unit cost benchmarks across a variety of settings.

For the tables of this paper, we generated country-specific benchmarks for both economic and financial unit costs. We set population treated (*pop*) to 10 thousand, 100 thousand and 1 million people respectively; the school-based (*sch*) dummy to 0; the national programme (*nat*) dummy to 1; the integrated delivery (*int*), years (*yrs*) and rounds (*rds*) variables to 1; the coverage (*cov*) and population density (*den*) variables to the sample medians (85% and 134 respectively). We set GDP per capita in 2015 US$ (*gdp*) to country-specific values, but constrained it to the maximum and minimum of the sample (2015 US$ 466 and 3737 respectively) to avoid extrapolating too far outside of the available data. We set the local volunteer (*vol*) dummy to 1 for financial benchmarks (resulting in lower cost) and to 0 for economic benchmarks (resulting in higher cost). To be clear, all unit cost benchmarks reported in this study exclude the cost of volunteer time. However, the economic unit cost benchmarks assume that volunteers are not used (i.e., that all labour inputs are paid).

A web-based software application was developed using *shiny* (RStudio) to calculate setting-specific unit costs against which programme budgets and expenditures or results-based pay-outs can be benchmarked. In the software application, all of the above parameters can be chosen by the user.

The logarithmic transformation of the unit cost benchmark (ucb) was obtained with the vector of coefficients (B) and the matrix of new values for the explanatory variables (X) and

log⁡(ucb)=BX′

The standard error of the (log) estimate was calculated using

$$SEE=\sqrt{XVX\prime }$$

where V is the variance-covariance matrix.

With the mean and standard error, we randomly drew from a normal distribution and re-transformed (exponentiated) 10 000 values and extracted the mean, 2.5th and 97.5th centile values for the best estimates and 95% Confidence Intervals (CIs). A 95% CI means that if the data are resampled, best estimates are expected to fall within this interval in 95% of the samples.

In the software application, we also provide prediction intervals (PIs). The standard error of the (log) prediction becomes

$$SEP=\sqrt{SSE/(n-2)+XVX\prime }$$

where SSE is the sum of squared errors (residuals).

A 95% PI means that if a single observation is resampled, the unit cost is expected to fall within this interval in 95% of the samples.

## Results

### Availability of Studies

**Fig 1:**
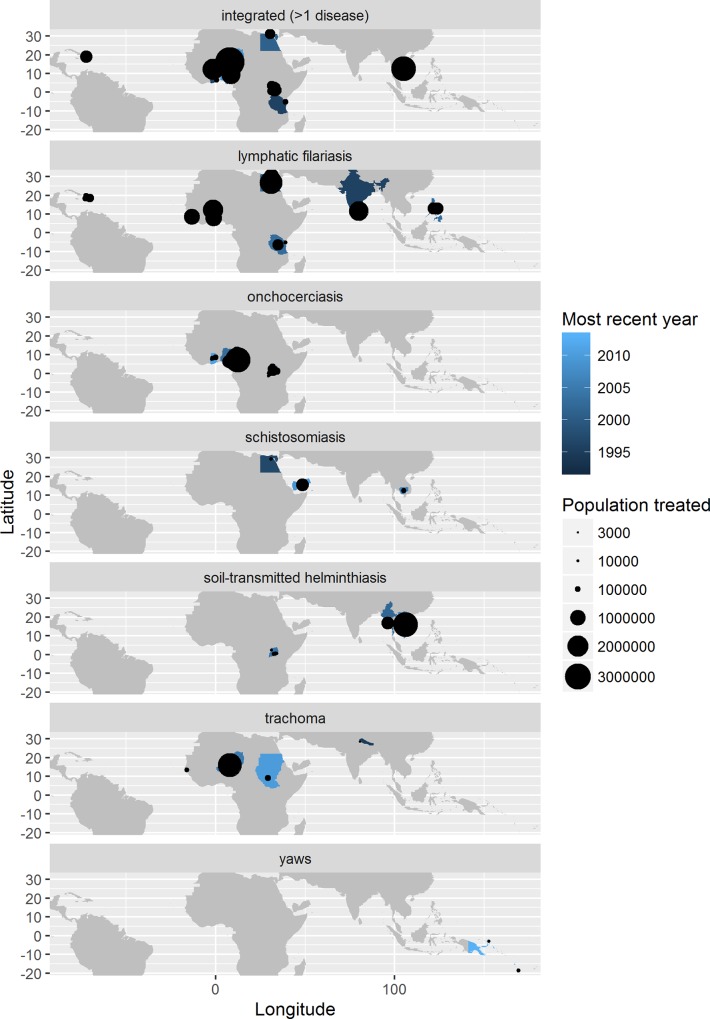
Availability of costing studies among low- and middle-income countries, by disease. Most recent year refers to the most recent year of study, not the most recent year of publication.

The 34 studies included in the meta-regression cover 21 countries and 91 sites over 19 years for a total of 212 different observations of unit cost. The countries and sites from which the studies were taken are depicted, by disease, in Fig 1. A disproportionate number of observations are from Uganda (96).

There are 150 observations of financial cost from 29 studies and 130 observations of economic cost from 17 studies, with 12 studies reporting both (Table 2). Financial unit costs (excluding medicines) range from US$ 0.01 per treatment to US$ 8.50, with a median value of $0.20. Economic unit costs (excluding medicines and volunteer time) ranged from $0.02 to $2.90, with a median value of $0.40.

**Table 2:** Summary statistics for 34 studies of 23 countries and 91 sites over 19 years

Statistic^1^	N	Mean	St. Dev.	Min	Pctl(25)	Median	Pctl(75)	Max
year	212	2003.3	4.3	1992	2000	2003	2007	2013
ucf	150	0.4	0.8	0.01	0.1	0.2	0.4	8.5
uce	130	0.7	0.6	0.02	0.3	0.4	1.0	2.9
vol	212	0.9	0.3	0	1	1	1	1
int	212	2.0	1.5	1	1	1	2	5
rds	212	1.2	0.5	1.0	1.0	1.0	1.0	3.0
yrs	212	4.0	2.9	1	2	3	5	11
cov	212	82.4	14.1	38	73.2	85	95	100
sch	212	0.2	0.4	0	0	0	0	1
pop	212	314,687.6	596,443.9	500	38,059	116,815.5	272,868	3,991,392
den	212	637.7	2,171.0	10.0	67.0	134.0	314.0	14,897.0
nat	212	0.2	0.4	0	0	0	0	1
gdp	212	739.4	624.8	138.5	359.6	466.4	810.0	3,737.7
dis	172	255.9	1,426.7	0	0	102	177.5	16,277

^*1*^ Where: *ucf* is financial unit cost in 2015 US$, not reported in all studies; *uce* is economic unit cost in 2015 US$, not reported in all studies; *vol* is the volunteer dummy for use of local volunteers (not costed); *int* is the number of diseases for which delivery is integrated; *rds* is the number of rounds per year; *yrs* is the number of years of programme implementation; *cov* is the percentage coverage achieved; *sch* is the school-based dummy for treatment of school children only; *pop* is the total number of people treated; *den* is population density per km^2^; *gdp* is GDP per capita (2015 US$); *den* is population density (people per km^2^); *dis* is the distance in km from the study site to the nearest city of >100 000 inhabitants, only for those studies that were not national.

Most studies are from low-income settings with 90% of observations from studies reporting the use of volunteers and 20% are from studies of school-based programmes. The average (median) observation is 116 816 people treated for 2 diseases over one round in the 3^rd^ year of programme implementation. Median coverage was 85% overall, which was the same for the school-based programmes subset. Median population density was 134 people per km^2^; among the subset of subnational sites, the average population density was slightly higher (142 people per km^2^). Subnational sites were located an average (median) of 102 km from the nearest city of >100 000 inhabitants.

**Fig 2:**
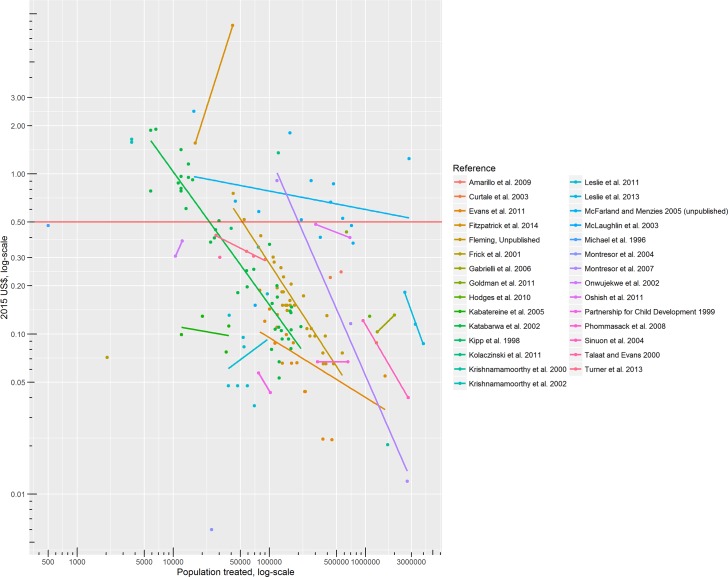
Financial unit cost and population treated, by study (across years, sites and comparators). Dots represent individual study results, and lines represent the least squares line of best fit for studies with more than two results. The horizontal line at US$ 0.50 marks the oft-cited unit cost typically used in advocacy.

**Fig 3:**
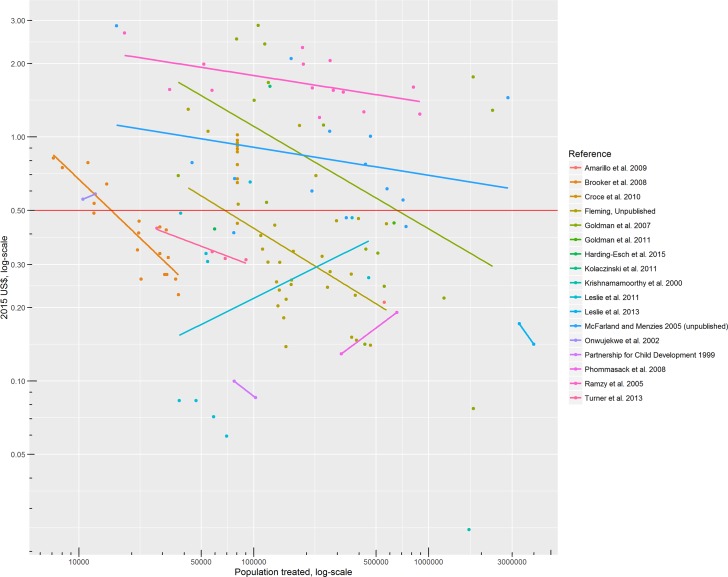
Economic unit costs (excluding volunteer time) and population treated, by study (across years, sites and comparators). Dots represent individual study results, and lines represent the least squares line of best fit for studies with more than two results. The horizontal line at US$ 0.50 marks the oft-cited unit cost typically used in advocacy.

Fig 2 plots financial unit costs against populations treated, with each colour representing a different study. The lines show the log-log within-study relationship between unit costs and population treated. Similarly, Fig 3 shows the log-log within-study relationship between economic unit costs and population treated.

There are a number of clear outliers. Three studies stand out for their low estimates of cost, describing fewer cost categories than the others.[8][9][10] To the random effects regression model, we add dummy variables for these three studies. One study stands out for its high estimate of cost. In Tafea province (Vanuatu), the financial cost of TCT (for yaws) of 41 509 people distributed over five remote islands with very weak health and road infrastructure was about than 2015 US$ 8.47 per person.[11] To both the random and fixed effects regression models, therefore, we added a dummy variable for Vanuatu, a small island developing state (SIDS).

### Predictors of Unit Cost

Regression model results for unit costs in 2015 US$ are presented in Table 3.

**Table 3:** Results from meta-regression of (log) unit costs in 2015 US$

	*Dependent variable*:	
	log(ucb)	
	(1)	(2)
eco	0.157 (0.122)	0.178 (0.124)
vol	-1.348*** (0.288)	-0.830* (0.436)
log(int)	-0.170 (0.306)	-0.544 (0.630)
log(rds)	-0.260** (0.130)	-0.222* (0.125)
yrs	-0.007 (0.020)	-0.003 (0.022)
cov	-0.007** (0.003)	-0.008** (0.003)
nat	3.667* (2.097)	
sch	2.266*** (0.563)	2.121*** (0.538)
log(pop)	-0.527*** (0.037)	-0.545*** (0.036)
log(den)	0.080** (0.041)	0.082* (0.042)
log(gdp)	0.738*** (0.162)	0.880*** (0.170)
VUT	1.787*** (0.612)	1.921*** (0.586)
Kri	-2.285** (1.038)	-1.641 (1.460)
Fri	-2.322** (1.076)	-2.174 (1.475)
Mon	-2.616** (1.115)	-2.860* (1.513)
sqrt(dis)		0.003 (0.005)
eco:log(int)	0.348*** (0.105)	0.339*** (0.103)
cov:nat	0.016 (0.014)	
eco:sch	0.776*** (0.224)	0.765*** (0.237)
cov:sch	-0.025*** (0.007)	-0.019*** (0.007)
nat:log(pop)	0.041 (0.105)	
nat:log(den)	-0.640*** (0.190)	
nat:log(gdp)	-0.291 (0.247)	
Constant	1.134 (1.179)	-0.140 (1.325)
Observations	280	232
R^2^	0.665	0.677
Adjusted R^2^	0.610	0.622
F Statistic	23.127*** (df = 22; 257)	24.760*** (df = 18; 213)

Note

*p<0.1

**p<0.05

***p<0.01

Refer to Table 2 or Methods for a brief description of the variables. Additionally, *VUT is a* dummy for Vanuatu. *Fri*, *Kri* and *Mon* are dummies for three studies with incomplete cost categories. [8][9][10] Colons (:) indicated interaction terms.

There is a significant and strongly negative association between unit costs and the number of people treated, confirming the expectation of important economies of scale. Similarly, an increase in the number of rounds per year is also associated with a significantly lower unit cost per person treated per round, suggesting that fixed (annual) costs can be shared across rounds too. Use of local (village or school) volunteers is associated with significantly lower unit costs, both financial and economic (including the cost of Ministry of Health staff time and assets but excluding the economic cost of volunteer time).

Population density (meant to capture logistical ease of access) is negatively associated with unit costs among national programmes, but positively associated with unit costs among subnational programmes. Among these subnational sites, distance from the nearest city (meant to capture logistical difficulty) does not turn out to be associated with unit costs.

Integrated delivery of medicines is not associated with higher financial unit costs, but is associated with higher economic unit costs, suggesting that there may be some coordination costs related to integration.

Overall, unit costs are higher in national programmes, implying some diseconomies of scale as geographic coverage moves from subnational sites to national programmes covering more diverse settings. However, among subnational sites, the association between unit costs and programme coverage is negative, especially in the case of school-based programmes. School-based programmes are associated with higher unit costs after controlling for coverage and the use of local volunteers; all school-based programmes benefited from high coverage (enrolment) and use of volunteers.

GDP per capita is positively associated with unit cost, capturing (at least in part) the quality and complexity of inputs. The number of years or programme implementation (meant to capture any learning-by-doing effects) is not significantly associated with unit cost, but the (negative) sign of the coefficient is as expected. Unit costs are very much higher in Vanuatu, possibly reflecting the higher cost of implementation in a SIDS, and very much lower in the three studies which we deemed to be incomplete in terms of cost categories.

Since the distance variable (subnational data) is not statistically significant, we proceed in what follows with random effects Model 1 (based on the full set of data). The R^2^ statistic suggests that it explains about two thirds of the variation in unit costs reported in the literature. Transforming unit costs into PPP dollars does not much improve the explanatory power of the model. We therefore remained with the arguably more easily and widely understood estimates based on unit costs in US$. Results in PPP dollars are nonetheless presented with the fixed effects model results in the Supplemental Information (S4 Table).

### Unit Cost Benchmarks

**Fig 4:**
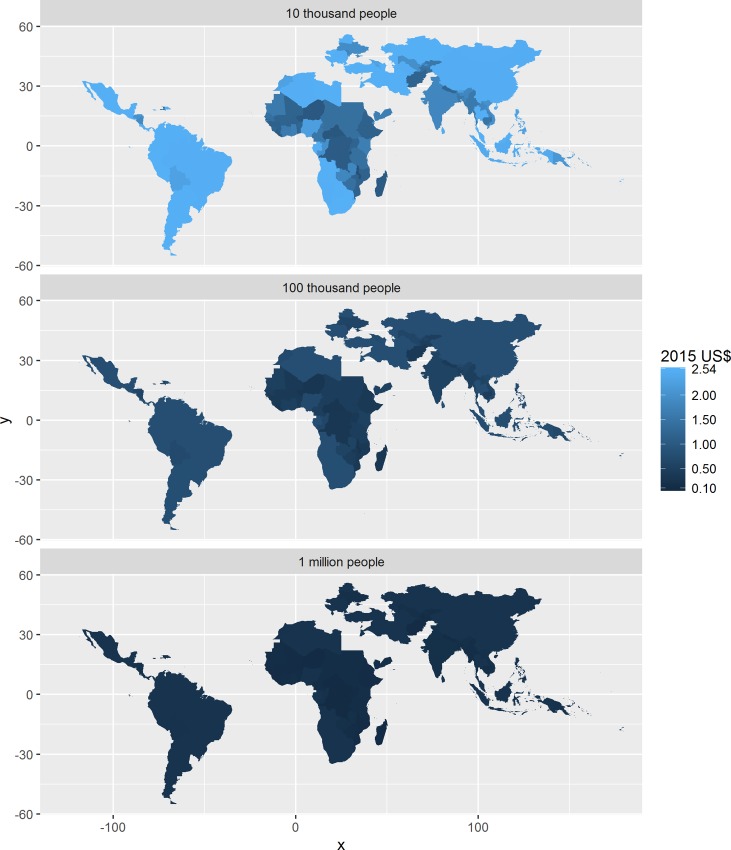
Financial unit cost benchmarks in low- and middle-income countries at different scales of implementation, using volunteers but excluding the (economic) cost of volunteer time. The legend excludes Vanuatu. See S5 Table in the Supplemental Information for results for Vanuatu.

Benchmarks for the financial unit cost in US$ are depicted in Fig 4 for all low and middle income countries, at three different scales of implementation. For programmes treating 100 000 people or more, the financial unit cost benchmark is less than US$ 0.50 for the vast majority of countries. However, the benchmark can exceed US$ 2.00 for programmes operating at a scale of about 10 000 people.

**Fig 5:**
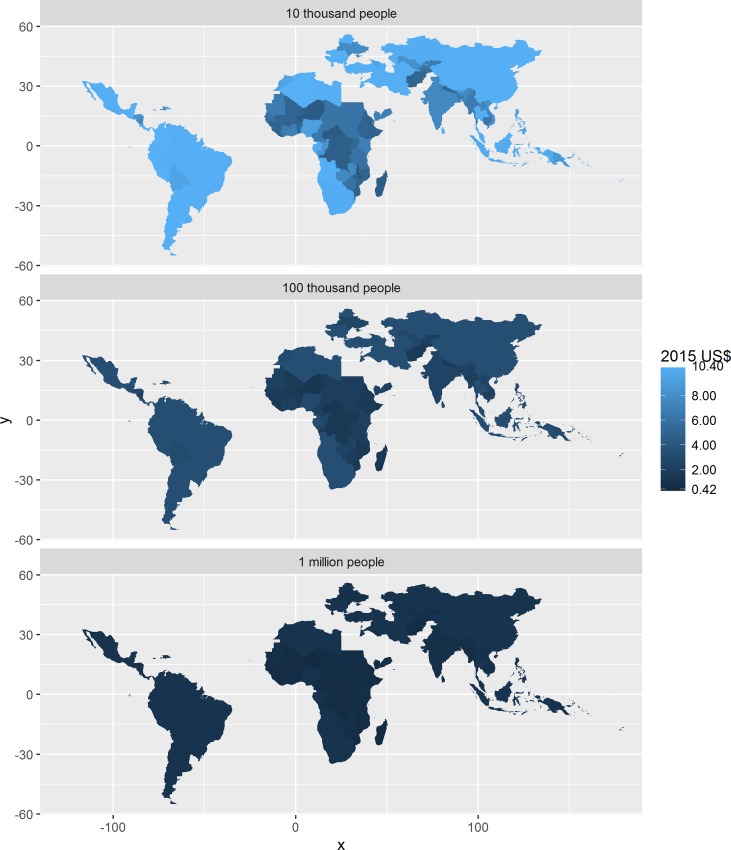
Economic unit cost benchmarks in low- and middle-income countries1 at different scales of implementation, not using volunteers. The legend excludes Vanuatu. See S5 Table in the Supplemental Information for results for Vanuatu.

Similarly, benchmarks for the economic unit cost in US$ are depicted in Fig 5. For programmes treating 100 000 people or more, the economic unit cost benchmark is less than US$ 1.00 for the vast majority of countries. However, the benchmark can exceed US$ 10.00 for programmes operating at a scale of about 10 000 people.

Benchmarks for both financial and economic unit costs are presented in data tables with 95% confidence intervals in the Supplemental Information (S5 Table). These regression results were used to create a web-based software application available at https://healthy.shinyapps.io/benchmark/. Users can enter setting-specific parameter values to arrive at a benchmark unit cost for their setting. These benchmarks can be compared by researchers to individual unit cost estimates extracted from the studies. A guide to using the application can be found in Supplemental Information available within the application at the abovementioned link.

All unit cost benchmarks reported by the web-based application exclude the opportunity cost of local volunteer time. However, one can choose to assume that volunteers are not used for the mass treatment campaigns, in which case the unit cost benchmarks are estimated as though all labour inputs are paid at their respective wages. This is true whether one chooses the random effects or the fixed effects model.

## Discussion

This study provides the most up-to-date review of the literature on the cost of mass treatment for the control and elimination of six selected NTDs for which mass treatment is recommended by WHO. For the first time, the evidence has been standardized and synthesized in a meta-regression of the cost per person treated per round, controlling for differences between settings. We find that unit costs are very sensitive to economies of scale, and the decision on whether or not to use local volunteers. Financial unit costs are expected to be less than 2015 US$ 0.50 in most countries with programmes that treat 100 000 people or more. However, for smaller programmes, including those in the “last mile”, or those that cannot rely on volunteers, financial unit costs may be considerably higher.

Some of the regression results are surprising. Among subnational project sites, we do not find the expected significant positive association between unit cost and coverage nor between unit costs and population density or distance from the nearest city. These surprising results warrant further investigation; there may be better measures of logistical ease/difficulty of access.

This study then uses the meta-regression results to predict unit costs across a large number of different possible settings. One of the advantages of this approach over taking a simple arithmetic average across (subsets of) the available data is that it also gives robust confidence intervals. The confidence intervals can be used in economic evaluations, including cost-effectiveness analyses looking to generalize results across settings, but are not meant to be used as substitutes for detailed planning and budgeting. However, confidence intervals can be used to assess value for money in programme plans, budgets and accounts, or help set a reasonable pay-out for results-based financing mechanisms.

The approach can be used for other outreach interventions, beyond health care facilities, that may gain in importance in the context of universal health coverage. Indeed, our benchmarks may be relevant for intermittent preventive treatment (IPT) against malaria. IPT involves a full course of an anti-malarial treatment in areas of seasonal transmission, regardless of individual infection. In Ghana, the economic cost was at least US$ 2.35 (2008) per month of intervention for a group of 613 children that received IPT.[12]

In future, unit costs of mass treatment against NTDs could be benchmarked against the unit cost of other mass interventions. A review of the cost of vitamin A supplementation suggests that unit costs can vary by a factor of more than 1000 and are several-fold higher than has traditionally been maintained.[13] Our benchmarks are unlikely to be of relevance to mass immunization requiring a costly cold chain. However, they could give an indication of cost savings associated with the integration of mass treatment against NTDs within existing immunization campaigns.[14]

There are a few caveats. For one, confidence and prediction intervals are wide and even so do not fully reflect true uncertainty. While there are a good number of studies available, they do not cover as many countries as there are studies. Most report only financial costs, excluding the cost of Ministry of Health resources (buildings and staff), for example, and therefore fail to estimate the full costs to the health system. Many appear to be from peri-urban areas rather than from the rural areas in which most of the population requiring treatment is found or indeed from the remotest areas where one would expect unit costs to be highest.

Another limitation is that while most programmes used volunteers, few studies considered the economic cost of their time. We controlled for the use of volunteers in the regression model and estimate that, all other things being equal, the financial cost more than doubles in going from volunteers to paid health workers. Unfortunately, the use of unpaid volunteers may not be scalable: “fully-scaled NTD control programmes covering over a billion people cannot expect to recruit and retain sufficient numbers of volunteers if other major disease programmes are offering incentives.”[1]

Furthermore, while the focus continues to shift from control to elimination, most of the available costing studies are of control programmes. Of the 34 studies identified in our review, only 8 referred explicitly to eradication (yaws) or elimination (LF, onchocerciasis) as a programme objective.[11][15][16][17][18][19][20][21] Only one of those directly compared the costs of control and elimination strategies (for onchocerciasis), involving annual and biannual (twice yearly) mass treatment respectively; the difference is determined by the number of rounds rather than by so-called “last mile” costs.[21]

No study has been conducted in a country where eradication or elimination has actually been achieved. Egypt stopped PC and started post-PC surveillance for LF in 2014, but the available costs are from 2000–2001. Challenges to elimination posed by the parasite *Loa loa*, that can cause fatal side-effects upon anthelmintic treatment, have not been factored into any of the available costing studies of LF, and into only one country from one study of onchocerciasis.[3]

Finally, most of the available costing studies consider the early years of programme implementation. Some considered the cost of planning and mapping in these early years, but few considered longer-term monitoring and evaluation. A micro-costing study based on other sources (and therefore excluded from the meta-regression) estimates that the financial unit cost per treatment would increase two times towards the later phases of elimination of onchocerciasis in Africa.[22] This increase is driven by the reduction in the number of people in need of treatment and steady or increasing costs for surveillance.

Klepac et al (2015) provide a good summary of why the distinction between the “middle game” and the “endgame” matters.[23] First, the endgame is associated with higher unit costs; the last foci of infection or pockets of susceptibility will be those that are hardest to reach, either geographically or socially (e.g. treatment refusers). Second, the endgame may present fewer opportunities for cost-sharing across interventions; while elimination can and should continue to be delivered by strong health systems, frequency and timing become less flexible in the endgame.

The duration and total cost of the endgame is likely to be a function of: “the underlying biology of the pathogen, the demography of the host(s), the connectedness of affected populations, the speed of roll out of control measures, their efficacy and the capacity for sustained effort, likely to be itself shaped by political agendas and financing.”[24] A prolonged and expensive endgame can lead to funder fatigue and motivate a (premature) switch in strategy from, for example, mass treatment to targeted treatment in remaining foci of infection or high-risk locations or populations.

While our review of the literature, published and grey, was thorough, more could be done to identify more studies, such as: looking at more databases and considering other languages spoken in a small number of endemic countries, namely Arabic, Chinese and Portuguese. In future, country and technical expert groups could be convened to reconsider the data and approach used, similar to benchmarking work undertaken for the Global Fund to Fight AIDS, Tuberculosis and Malaria.

Other refinements to this study might include benchmarks for the cost of post-mass treatment mop-up, known more formally in the trachoma and yaws literature as “enhanced coverage” and “total targeted treatment”, respectively, in which communities are visited a second time to treat only those not treated on the first visit.[11][25] More evidence is needed on the cost of post-mass treatment surveillance, including Transmission Assessment Surveys, and certification of eradication or elimination. Indeed, we are not aware of any studies of the cost of eradication or elimination of diseases (including guinea-worm disease or poliomyelitis) that explicitly included the cost of the certification process.

This comprehensive analysis confirms that mass treatment offers a low cost public health intervention on the path towards universal health coverage. However, more costing studies focussed on elimination are needed. The novel web-based platform https://healthy.shinyapps.io/benchmark/ can be used to determine realistic unit cost benchmarks to assist monitoring value for money in NTD programme plans, budgets and accounts, or in setting a reasonable pay-out for results-based financing mechanisms by Ministries of Health and Finance in low- and middle-income countries.

## Supporting Information

S1 Checklist PRISMA 2009 checklist (PDF)

S1 Table Pubmed search terms (DOCX)

S2 Table Studies excluded from meta-regression (DOCX)

S3 Table Studies included in meta-regression (DOCX)

S4 Table Results from meta-regression using fixed effects model or unit costs in 2015 I$ (PPP) (DOCX)

S5 Table Benchmarks for financial and economic unit costs with 95% confidence intervals at different scales of implementation, 2015 US$ (DOCX)
