# Supplementary material for: Benchmarking the Cost per Person of Mass Treatment for Selected Neglected Tropical Diseases: An Approach Based on Literature Review and Meta-regression with Web-Based Software Application
Source: PLoS Negl Trop Dis. 2016 Dec 5;10(12):e0005037. doi: 10.1371/journal.pntd.0005037 (PMC5137870; doi:10.1371/journal.pntd.0005037)
Supplement: S1 Table — (DOCX) [file pntd.0005037.s002.docx]

**S1 Table. Pubmed search terms**

(filariasis[Title] OR onchocerciasis[Title] OR schistosomiasis[Title] OR helminthiases[Title] OR helminthiasis[Title] OR helminth[Title] OR helminths[Title] OR trachoma[Title] OR yaws[Title] OR “neglected tropical diseases”[Title] OR “neglected diseases”[Title] OR “neglected infectious diseases”[Title] OR ascariasis[Title] OR hookworm[Title] OR roundworm[Title] OR trichuriasis[Title] OR whipworm[Title] OR bilharziasis[Title] OR “snail fever”[Title] OR “river blindness” [Title] OR elephantiasis[Title] OR “preventive chemotherapy”[Title] OR “mass treatment”[Title] or “mass drug administration”[Title] OR deworming[Title] OR ivermectin[Title] OR mectizan[Title] OR albendazole[Title] OR mebendazole[Title] or praziquantel[Title]) AND (economic[Title] OR economics[Title] OR cost[Title] OR costs[Title] OR costing[Title] OR resource[Title] OR price[Title] OR expenditure[Title] OR spending[Title] OR fund[Title] OR funds[Title] OR funding[Title]) AND (("1990"[Date - Publication] : "3000"[Date - Publication]))
