# Supplementary material for: Benchmarking the Cost per Person of Mass Treatment for Selected Neglected Tropical Diseases: An Approach Based on Literature Review and Meta-regression with Web-Based Software Application
Source: PLoS Negl Trop Dis. 2016 Dec 5;10(12):e0005037. doi: 10.1371/journal.pntd.0005037 (PMC5137870; doi:10.1371/journal.pntd.0005037)
Supplement: S2 Table — (DOCX) [file pntd.0005037.s003.docx]

**S2 Table. Studies excluded from meta-regression**

1.

Baltussen RMPM, Sylla M, Frick KD, Mariotti SP. Cost-effectiveness of trachoma control in seven world regions. Ophthalmic Epidemiol. 2005;12: 91–101. doi:10.1080/09286580590932761

2.

Boselli G, Yajima A, Aratchige PE, Feldon KE, Xeuatvongsa A, Phounphenghak K, et al. Integration of deworming into an existing immunisation and vitamin A supplementation campaign is a highly effective approach to maximise health benefits with minimal cost in Lao PDR. Int Health. 2011;3: 240–245. doi:10.1016/j.inhe.2011.08.002

3.

Carabin H, Chan MS, Guyatt HL. A population dynamic approach to evaluating the impact of school attendance on the unit cost and effectiveness of school-based schistosomiasis chemotherapy programmes. Parasitology. 2000;121 ( Pt 2): 171–183.

4.

Coffeng LE, Stolk WA, Zouré HGM, Veerman JL, Agblewonu KB, Murdoch ME, et al. African Programme For Onchocerciasis Control 1995-2015: model-estimated health impact and cost. PLoS Negl Trop Dis. 2013;7: e2032. doi:10.1371/journal.pntd.0002032

5.

Colston J, Saboyá M. Soil-transmitted helminthiasis in Latin America and the Caribbean: modelling the determinants, prevalence, population at risk and costs of control at sub-national level. Geospat Health. 2013;7: 321–340. doi:10.4081/gh.2013.90

6.

De Rochars MB, Kanjilal S, Direny AN, Radday J, Lafontant JG, Mathieu E, et al. The Leogane, Haiti demonstration project: decreased microfilaremia and program costs after three years of mass drug administration. Am J Trop Med Hyg. 2005;73: 888–894.

7.

Evans DB, Guyatt HL. The cost effectiveness of mass drug therapy for intestinal helminths. Pharmacoeconomics. 1995;8: 14–22.

8.

Evans TG, Ranson MK, Kyaw TA, Ko CK. Cost effectiveness and cost utility of preventing trachomatous visual impairment: lessons from 30 years of trachoma control in Burma. Br J Ophthalmol. 1996;80: 880–889.

9.

Guyatt H, Evans D, Lengeler C, Tanner M. Controlling schistosomiasis: the cost-effectiveness of alternative delivery strategies. Health Policy Plan. 1994;9: 385–395.

10.

Guyatt H. The cost of delivering and sustaining a control programme for schistosomiasis and soil-transmitted helminthiasis. Acta Trop. 2003;86: 267–274.

11.

Guyatt HL, Bundy DA, Evans D. A population dynamic approach to the cost-effectiveness analysis of mass anthelmintic treatment: effects of treatment frequency on Ascaris infection. Trans R Soc Trop Med Hyg. 1993;87: 570–575.

12.

Guyatt HL. The economics of worm control. Kluwer Academic Press; 2002.

13.

Hall A, Horton S, de Silva N. The costs and cost-effectiveness of mass treatment for intestinal nematode worm infections using different treatment thresholds. PLoS Negl Trop Dis. 2009;3: e402. doi:10.1371/journal.pntd.0000402

14.

Holland CV, O’Shea E, Asaolu SO, Turley O, Crompton DW. A cost-effectiveness analysis of anthelminthic intervention for community control of soil-transmitted helminth infection: levamisole and Ascaris lumbricoides. J Parasitol. 1996;82: 527–530.

15.

Keating J, Yukich JO, Mollenkopf S, Tediosi F. Lymphatic filariasis and onchocerciasis prevention, treatment, and control costs across diverse settings: A systematic review. Acta Tropica. 2014;135: 86–95. doi:10.1016/j.actatropica.2014.03.017

16.

Montresor A, Gabrielli AF, Diarra A, Engels D. Estimation of the cost of large-scale school deworming programmes with benzimidazoles. Trans R Soc Trop Med Hyg. 2010;104: 129–132. doi:10.1016/j.trstmh.2009.10.007

17.

Ndeffo Mbah ML, Poolman EM, Atkins KE, Orenstein EW, Meyers LA, Townsend JP, et al. Potential cost-effectiveness of schistosomiasis treatment for reducing HIV transmission in Africa--the case of Zimbabwean women. PLoS Negl Trop Dis. 2013;7: e2346. doi:10.1371/journal.pntd.0002346

18.

Stolk WA, ten Bosch QA, de Vlas SJ, Fischer PU, Weil GJ, Goldman AS. Modeling the impact and costs of semiannual mass drug administration for accelerated elimination of lymphatic filariasis. PLoS Negl Trop Dis. 2013;7: e1984. doi:10.1371/journal.pntd.0001984

19.

Turner HC, Walker M, Attah SK, Opoku NO, Awadzi K, Kuesel AC, et al. The potential impact of moxidectin on onchocerciasis elimination in Africa: an economic evaluation based on the Phase II clinical trial data. Parasit Vectors. 2015;8: 167. doi:10.1186/s13071-015-0779-4

20.

Turner HC, Walker M, Churcher TS, Osei-Atweneboana MY, Biritwum N-K, Hopkins A, et al. Reaching the London Declaration on Neglected Tropical Diseases Goals for Onchocerciasis: An Economic Evaluation of Increasing the Frequency of Ivermectin Treatment in Africa. Clin Infect Dis. 2014; doi:10.1093/cid/ciu467

21.

Waters HR, Rehwinkel JA, Burnham G. Economic evaluation of Mectizan distribution. Trop Med Int Health. 2004;9: A16–25. doi:10.1111/j.1365-3156.2004.01210.x

22.

Zhou X-N, Wang L-Y, Chen M-G, Wang T-P, Guo J-G, Wu X-H, et al. An economic evaluation of the national schistosomiasis control programme in China from 1992 to 2000. Acta Trop. 2005;96: 255–265. doi:10.1016/j.actatropica.2005.07.026
