# Supplementary material for: Benchmarking the Cost per Person of Mass Treatment for Selected Neglected Tropical Diseases: An Approach Based on Literature Review and Meta-regression with Web-Based Software Application
Source: PLoS Negl Trop Dis. 2016 Dec 5;10(12):e0005037. doi: 10.1371/journal.pntd.0005037 (PMC5137870; doi:10.1371/journal.pntd.0005037)
Supplement: S3 Table — (DOCX) [file pntd.0005037.s004.docx]

**S3 Table. Studies included in meta-regression**

1.

Amarillo M, Belizario VJ, Panelo C, Sison S, de Leon W, Ramirez B, et al. Cost of mass drug administration for filariasis elimination in the province of Sorsogon, Philippines. Acta Med Philipp. 2009;43: 23–28.

2.

Brooker S, Kabatereine NB, Fleming F, Devlin N. Cost and cost-effectiveness of nationwide school-based helminth control in Uganda: intra-country variation and effects of scaling-up. Health Policy Plan. 2008;23: 24–35. doi:10.1093/heapol/czm041

3.

Croce D, Porazzi E, Foglia E, Restelli U, Sinuon M, Socheat D, et al. Cost-effectiveness of a successful schistosomiasis control programme in Cambodia (1995-2006). Acta Trop. 2010;113: 279–284. doi:10.1016/j.actatropica.2009.11.011

4.

Curtale F, Abd-el Wahab Hassanein Y, El Wakeel A, Barduagni P, Savioli L. The School Health Programme in Behera: an integrated helminth control programme at Governorate level in Egypt. Acta Trop. 2003;86: 295–307.

5.

Evans D, McFarland D, Adamani W, Eigege A, Miri E, Schulz J, et al. Cost-effectiveness of triple drug administration (TDA) with praziquantel, ivermectin and albendazole for the prevention of neglected tropical diseases in Nigeria. Ann Trop Med Parasitol. 2011;105: 537–547. doi:10.1179/2047773211Y.0000000010

6.

Fitzpatrick C, Asiedu K, Jannin J. Where the road ends, yaws begins? The cost-effectiveness of eradication versus more roads. PLoS Negl Trop Dis. 2014;8: e3165. doi:10.1371/journal.pntd.0003165

7.

Fleming F. (Unpublished).

8.

Frick KD, Lietman TM, Holm SO, Jha HC, Chaudhary JS, Bhatta RC. Cost-effectiveness of trachoma control measures: comparing targeted household treatment and mass treatment of children. Bull World Health Organ. 2001;79: 201–207.

9.

Gabrielli A-F, Touré S, Sellin B, Sellin E, Ky C, Ouedraogo H, et al. A combined school- and community-based campaign targeting all school-age children of Burkina Faso against schistosomiasis and soil-transmitted helminthiasis: performance, financial costs and implications for sustainability. Acta Trop. 2006;99: 234–242. doi:10.1016/j.actatropica.2006.08.008

10.

Goldman AS, Brady MA, Direny A, Desir L, Oscard R, Vely J-F, et al. Costs of integrated mass drug administration for neglected tropical diseases in Haiti. Am J Trop Med Hyg. 2011;85: 826–833. doi:10.4269/ajtmh.2011.10-0635

11.

Goldman AS, Guisinger VH, Aikins M, Amarillo MLE, Belizario VY, Garshong B, et al. National mass drug administration costs for lymphatic filariasis elimination. PLoS Negl Trop Dis. 2007;1: e67. doi:10.1371/journal.pntd.0000067

12.

Harding-Esch E, Jofre-Bonet M, Dhanjal JK, Burr S, Edwards T, Holland M, et al. Costs of testing for ocular Chlamydia trachomatis infection compared to mass drug administration for trachoma in the Gambia: application of results from the PRET study. PLoS Negl Trop Dis. 2015;9: e0003670. doi:10.1371/journal.pntd.0003670

13.

Hodges MH, Smith SJ, Fussum D, Koroma JB, Conteh A, Sonnie M, et al. High coverage of mass drug administration for lymphatic filariasis in rural and non-rural settings in the Western Area, Sierra Leone. Parasit Vectors. 2010;3: 120. doi:10.1186/1756-3305-3-120

14.

Kabatereine NB, Tukahebwa EM, Kazibwe F, Twa-Twa JM, Barenzi JFZ, Zaramba S, et al. Soil-transmitted helminthiasis in Uganda: epidemiology and cost of control. Trop Med Int Health. 2005;10: 1187–1189. doi:10.1111/j.1365-3156.2005.01509.x

15.

Katabarwa MN, Habomugisha P, Richards FO. Implementing community-directed treatment with ivermectin for the control of onchocerciasis in Uganda (1997-2000): an evaluation. Ann Trop Med Parasitol. 2002;96: 61–73. doi:10.1179/000349802125002419

16.

Kipp W, Burnham G, Bamuhiiga J, Weis P, Büttner DW. Ivermectin distribution using community volunteers in Kabarole district, Uganda. Health Policy Plan. 1998;13: 167–173.

17.

Kolaczinski JH, Robinson E, Finn TP. The cost of antibiotic mass drug administration for trachoma control in a remote area of South Sudan. PLoS Negl Trop Dis. 2011;5: e1362. doi:10.1371/journal.pntd.0001362

18.

Krishnamoorthy K, Rajendran R, Sunish IP, Reuben R. Cost-effectiveness of the use of vector control and mass drug administration, separately or in combination, against lymphatic filariasis. Ann Trop Med Parasitol. 2002;96 Suppl 2: S77–90. doi:10.1179/000349802125002428

19.

Krishnamoorthy K, Ramu K, Srividya A, Appavoo NC, Saxena NB, Lal S, et al. Cost of mass annual single dose diethylcarbamazine distribution for the large scale control of lymphatic filariasis. Indian J Med Res. 2000;111: 81–89.

20.

Leslie J, Garba A, Boubacar K, Yayé Y, Sebongou H, Barkire A, et al. Neglected tropical diseases: comparison of the costs of integrated and vertical preventive chemotherapy treatment in Niger. Int Health. 2013;5: 78–84. doi:10.1093/inthealth/ihs010

21.

Leslie J, Garba A, Oliva EB, Barkire A, Tinni AA, Djibo A, et al. Schistosomiasis and soil-transmitted helminth control in Niger: cost effectiveness of school based and community distributed mass drug administration [corrected]. PLoS Negl Trop Dis. 2011;5: e1326. doi:10.1371/journal.pntd.0001326

22.

McFarland D, Menzies N, Njoumemi Z, Onwujekwe O. Study of cost per treatment with ivermectin using the CDTI strategy. African Programme for Onchocerciasis Control (APOC); 2005.

23.

McLaughlin SI, Radday J, Michel MC, Addiss DG, Beach MJ, Lammie PJ, et al. Frequency, severity, and costs of adverse reactions following mass treatment for lymphatic filariasis using diethylcarbamazine and albendazole in Leogane, Haiti, 2000. Am J Trop Med Hyg. 2003;68: 568–573.

24.

Michael E, Meyrowitsch DW, Simonsen PE. Cost and cost effectiveness of mass diethylcarbamazine chemotherapy for the control of bancroftian filariasis: comparison of four strategies in Tanzania. Trop Med Int Health. 1996;1: 414–426.

25.

Montresor A, Cong DT, Le Anh T, Ehrhardt A, Mondadori E, Thi TD, et al. Cost containment in a school deworming programme targeting over 2.7 million children in Vietnam. Trans R Soc Trop Med Hyg. 2007;101: 461–464. doi:10.1016/j.trstmh.2006.07.008

26.

Montresor A, Zin TT, Padmasiri E, Allen H, Savioli L. Soil-transmitted helminthiasis in Myanmar and approximate costs for countrywide control. Trop Med Int Health. 2004;9: 1012–1015. doi:10.1111/j.1365-3156.2004.01297.x

27.

Onwujekwe O, Chima R, Shu E, Okonkwo P. Community-directed treatment with ivermectin in two Nigerian communities: an analysis of first year start-up processes, costs and consequences. Health Policy. 2002;62: 31–51.

28.

Oshish A, AlKohlani A, Hamed A, Kamel N, AlSoofi A, Farouk H, et al. Towards nationwide control of schistosomiasis in Yemen: a pilot project to expand treatment to the whole community. Trans R Soc Trop Med Hyg. 2011;105: 617–627. doi:10.1016/j.trstmh.2011.07.013

29.

Partnership for Child Development. The cost of large-scale school health programmes which deliver anthelmintics to children in Ghana and Tanzania. The Partnership for Child Development. Acta Trop. 1999;73: 183–204.

30.

Phommasack B, Saklokham K, Chanthavisouk C, Nakhonesid-Fish V, Strandgaard H, Montresor A, et al. Coverage and costs of a school deworming programme in 2007 targeting all primary schools in Lao PDR. Trans R Soc Trop Med Hyg. 2008;102: 1201–1206. doi:10.1016/j.trstmh.2008.04.036

31.

Ramzy RMR, Goldman AS, Kamal HA. Defining the cost of the Egyptian lymphatic filariasis elimination programme. Filaria J. 2005;4: 7. doi:10.1186/1475-2883-4-7

32.

Sinuon M, Tsuyuoka R, Socheat D, Montresor A, Palmer K. Financial costs of deworming children in all primary schools in Cambodia. Trans R Soc Trop Med Hyg. 2005;99: 664–668. doi:10.1016/j.trstmh.2004.12.004

33.

Talaat M, Evans DB. The costs and coverage of a strategy to control schistosomiasis morbidity in non-enrolled school-age children in Egypt. Trans R Soc Trop Med Hyg. 2000;94: 449–454.

34.

Turner HC, Osei-Atweneboana MY, Walker M, Tettevi EJ, Churcher TS, Asiedu O, et al. The cost of annual versus biannual community-directed treatment of onchocerciasis with ivermectin: Ghana as a case study. PLoS Negl Trop Dis. 2013;7: e2452. doi:10.1371/journal.pntd.0002452
