# Supplementary material for: Benchmarking the Cost per Person of Mass Treatment for Selected Neglected Tropical Diseases: An Approach Based on Literature Review and Meta-regression with Web-Based Software Application
Source: PLoS Negl Trop Dis. 2016 Dec 5;10(12):e0005037. doi: 10.1371/journal.pntd.0005037 (PMC5137870; doi:10.1371/journal.pntd.0005037)
Supplement: S4 Table — (DOCX) [file pntd.0005037.s005.docx]

**S4 Table. Results from meta-regression using fixed effects model or unit costs in 2015 I$ (PPP)**

|  | | | | |
| --- | --- | --- | --- | --- |
|  | *Dependent variable:* | | | |
|  |  | | | |
|  | log(ucb) | | log(ucb * ppp) | |
|  | (1) | (2) | (3) | (4) |
|  | | | | |
| eco | 0.135 (0.124) | 0.164 (0.129) | 0.152 (0.121) | 0.179 (0.127) |
| vol | -1.484^***^ (0.335) | -0.792 (0.535) | -1.416^***^ (0.294) | -0.922^**^ (0.450) |
| log(int) | -0.451 (0.460) |  | -0.440 (0.327) | -0.640 (0.670) |
| log(rds) | -0.218^*^ (0.131) | -0.216^*^ (0.129) | -0.276^**^ (0.129) | -0.248^*^ (0.127) |
| yrs | -0.006 (0.020) | -0.004 (0.023) | -0.001 (0.020) | 0.006 (0.022) |
| cov | -0.006^*^ (0.003) | -0.007^**^ (0.003) | -0.007^**^ (0.003) | -0.008^**^ (0.003) |
| nat | 5.316^**^ (2.381) |  | 2.279 (2.130) |  |
| sch | 2.318^***^ (0.567) | 2.113^***^ (0.557) | 2.254^***^ (0.558) | 2.102^***^ (0.549) |
| log(pop) | -0.569^***^ (0.039) | -0.554^***^ (0.038) | -0.520^***^ (0.037) | -0.528^***^ (0.037) |
| log(den) | 0.089^**^ (0.043) | 0.085^*^ (0.045) | 0.082^**^ (0.041) | 0.085^**^ (0.043) |
| log(gdp) | 0.859^***^ (0.199) | 0.910^***^ (0.194) | 0.379^**^ (0.168) | 0.473^***^ (0.174) |
| VUT | 1.943^***^ (0.628) | 2.023^***^ (0.624) | 1.635^***^ (0.609) | 1.779^***^ (0.599) |
| sqrt(dis) |  | 0.004 (0.006) |  | 0.003 (0.006) |
| Kri |  |  | -2.244^*^ (1.186) | -1.665 (1.551) |
| Fri |  |  | -2.278^*^ (1.218) | -2.130 (1.566) |
| Mon |  |  | -2.507^**^ (1.254) | -2.762^*^ (1.603) |
| eco:log(int) | 0.360^***^ (0.105) | 0.349^***^ (0.106) | 0.349^***^ (0.104) | 0.339^***^ (0.105) |
| cov:nat | 0.008 (0.018) |  | 0.021 (0.014) |  |
| eco:sch | 0.838^***^ (0.226) | 0.766^***^ (0.246) | 0.792^***^ (0.222) | 0.757^***^ (0.242) |
| cov:sch | -0.022^***^ (0.007) | -0.017^**^ (0.007) | -0.024^***^ (0.007) | -0.018^**^ (0.007) |
| nat:log(pop) | 0.052 (0.110) |  | 0.044 (0.105) |  |
| nat:log(den) | -0.846^***^ (0.212) |  | -0.606^***^ (0.193) |  |
| nat:log(gdp) | -0.343 (0.289) |  | -0.150 (0.253) |  |
| Constant |  |  | 4.377^***^ (1.216) | 3.316^**^ (1.365) |
|  | | | | |
| Observations | 280 | 232 | 280 | 232 |
| R^2^ | 0.674 | 0.675 | 0.642 | 0.655 |
| Adjusted R^2^ | 0.546 | 0.558 | 0.589 | 0.601 |
| F Statistic | 24.661^***^ (df = 19; 227) | 28.460^***^ (df = 14; 192) | 20.885^***^ (df = 22; 257) | 22.375^***^ (df = 18; 213) |
|  | | | | |
| *Note:* | ^*^p<0.1; ^**^p<0.05; ^***^p<0.01 | | | |

Models 1 and 2 are fixed effects models of unit cost in 2015 US$. Models 3 and 4 are random effects models of unit cost in international dollars (I$), adjusted for purchasing power parity (PPP). Refer to Table 2 or Methods for a brief description of the variables. Additionally, *VUT is a* dummy for Vanuatu. *Fri, Kri* and *Mon* are dummies for three studies with incomplete cost categories. [8][9][10] Colons (:) indicated interaction terms.
