# Supplementary material for: Benchmarking the Cost per Person of Mass Treatment for Selected Neglected Tropical Diseases: An Approach Based on Literature Review and Meta-regression with Web-Based Software Application
Source: PLoS Negl Trop Dis. 2016 Dec 5;10(12):e0005037. doi: 10.1371/journal.pntd.0005037 (PMC5137870; doi:10.1371/journal.pntd.0005037)
Supplement: S5 Table — (DOCX) [file pntd.0005037.s006.docx]

**S5 Table. Benchmarks for financial and economic unit costs with 95% confidence intervals at different scales of implementation, 2015 US$**

| **Country** | **ISO3** | **gdp^1^** | **ppp^2^** | **Financial** | | | | | | **Economic** | | | | | |
| --- | --- | --- | --- | --- | --- | --- | --- | --- | --- | --- | --- | --- | --- | --- | --- |
|  |  |  |  | **10 thousand people** | | **100 thousand people** | | **1 million people** | | **10 thousand people** | | **100 thousand people** | | **1 million people** | |
| Afghanistan | AFG | 673 | 2.9 | 0.34 | (0.12-0.78) | 0.1 | (0.05-0.19) | 0.03 | (0.02-0.06) | 1.56 | (0.47-3.94) | 0.49 | (0.2-1.02) | 0.16 | (0.06-0.32) |
| Albania | ALB | 4495 | 2.6 | 0.86 | (0.22-2.32) | 0.26 | (0.09-0.57) | 0.08 | (0.03-0.17) | 3.77 | (1.07-9.45) | 1.15 | (0.47-2.37) | 0.37 | (0.17-0.7) |
| Algeria | DZA | 4742 | 3.1 | 0.74 | (0.2-1.99) | 0.23 | (0.08-0.49) | 0.07 | (0.03-0.15) | 3.31 | (0.95-8.26) | 0.99 | (0.41-2.05) | 0.32 | (0.15-0.6) |
| Angola | AGO | 4227 | 1.7 | 1.31 | (0.34-3.61) | 0.4 | (0.15-0.86) | 0.13 | (0.05-0.25) | 5.77 | (1.65-14.74) | 1.74 | (0.71-3.6) | 0.56 | (0.26-1.06) |
| Argentina | ARG | 13271 | 1.7 | 1.34 | (0.35-3.61) | 0.4 | (0.15-0.88) | 0.13 | (0.05-0.27) | 5.95 | (1.69-15.23) | 1.81 | (0.74-3.67) | 0.58 | (0.27-1.09) |
| Armenia | ARM | 2819 | 2.6 | 0.77 | (0.22-1.97) | 0.23 | (0.09-0.47) | 0.07 | (0.03-0.14) | 3.36 | (1-8.32) | 1.02 | (0.44-2.01) | 0.33 | (0.16-0.6) |
| Azerbaijan | AZE | 6576 | 2.7 | 0.85 | (0.22-2.28) | 0.26 | (0.09-0.56) | 0.08 | (0.03-0.16) | 3.71 | (1.05-9.5) | 1.13 | (0.47-2.35) | 0.36 | (0.17-0.68) |
| Bangladesh | BGD | 1284 | 2.8 | 0.48 | (0.16-1.13) | 0.15 | (0.07-0.27) | 0.05 | (0.02-0.08) | 2.2 | (0.68-5.32) | 0.67 | (0.29-1.32) | 0.22 | (0.1-0.4) |
| Belarus | BLR | 6425 | 2.8 | 0.82 | (0.22-2.18) | 0.25 | (0.09-0.53) | 0.08 | (0.03-0.16) | 3.6 | (1.03-9.16) | 1.1 | (0.46-2.23) | 0.35 | (0.16-0.66) |
| Belize | BLZ | 4834 | 1.7 | 1.32 | (0.34-3.62) | 0.4 | (0.15-0.88) | 0.13 | (0.06-0.26) | 5.81 | (1.67-15.02) | 1.76 | (0.73-3.6) | 0.57 | (0.27-1.08) |
| Benin | BEN | 767 | 2.5 | 0.41 | (0.14-0.95) | 0.13 | (0.06-0.23) | 0.04 | (0.02-0.07) | 1.93 | (0.58-4.84) | 0.59 | (0.24-1.22) | 0.19 | (0.08-0.39) |
| Bhutan | BTN | 2884 | 2.8 | 0.71 | (0.2-1.81) | 0.21 | (0.09-0.43) | 0.07 | (0.03-0.13) | 3.11 | (0.93-7.93) | 0.95 | (0.41-1.89) | 0.31 | (0.15-0.56) |
| Bolivia (Plurinational State of) | BOL | 3056 | 2.1 | 0.98 | (0.27-2.54) | 0.3 | (0.12-0.64) | 0.1 | (0.04-0.19) | 4.38 | (1.28-11.15) | 1.32 | (0.57-2.66) | 0.42 | (0.2-0.78) |
| Bosnia and Herzegovina | BIH | 4307 | 2.4 | 0.96 | (0.25-2.64) | 0.29 | (0.11-0.64) | 0.09 | (0.04-0.18) | 4.22 | (1.21-10.78) | 1.29 | (0.54-2.64) | 0.41 | (0.2-0.78) |
| Botswana | BWA | 7407 | 2.2 | 1.01 | (0.27-2.8) | 0.3 | (0.11-0.66) | 0.1 | (0.04-0.2) | 4.42 | (1.27-11.3) | 1.35 | (0.56-2.74) | 0.43 | (0.21-0.83) |
| Brazil | BRA | 9312 | 1.7 | 1.33 | (0.34-3.57) | 0.41 | (0.15-0.88) | 0.13 | (0.05-0.26) | 5.84 | (1.64-15.14) | 1.78 | (0.74-3.65) | 0.57 | (0.27-1.08) |
| Bulgaria | BGR | 7194 | 2.5 | 0.89 | (0.23-2.41) | 0.27 | (0.1-0.6) | 0.09 | (0.04-0.18) | 3.95 | (1.13-9.97) | 1.2 | (0.49-2.43) | 0.38 | (0.18-0.72) |
| Burkina Faso | BFA | 631 | 2.7 | 0.35 | (0.12-0.79) | 0.11 | (0.05-0.2) | 0.03 | (0.02-0.06) | 1.64 | (0.48-4.13) | 0.51 | (0.2-1.08) | 0.16 | (0.07-0.34) |
| Burundi | BDI | 360 | 2.6 | 0.29 | (0.1-0.66) | 0.09 | (0.04-0.17) | 0.03 | (0.01-0.05) | 1.37 | (0.36-3.72) | 0.43 | (0.14-0.99) | 0.14 | (0.05-0.32) |
| Cabo Verde | CPV | 3433 | 1.9 | 1.15 | (0.3-3.02) | 0.35 | (0.13-0.76) | 0.11 | (0.05-0.22) | 5.05 | (1.48-12.78) | 1.55 | (0.66-3.13) | 0.5 | (0.23-0.93) |
| Cambodia | KHM | 1146 | 3 | 0.42 | (0.14-0.98) | 0.13 | (0.06-0.24) | 0.04 | (0.02-0.07) | 1.91 | (0.58-4.58) | 0.59 | (0.25-1.16) | 0.19 | (0.09-0.36) |
| Cameroon | CMR | 1247 | 2.5 | 0.53 | (0.18-1.25) | 0.16 | (0.08-0.3) | 0.05 | (0.03-0.09) | 2.44 | (0.77-5.82) | 0.75 | (0.33-1.49) | 0.24 | (0.11-0.46) |
| Central African Republic | CAF | 376 | 1.7 | 0.46 | (0.15-1.05) | 0.14 | (0.07-0.27) | 0.05 | (0.02-0.09) | 2.16 | (0.58-5.68) | 0.66 | (0.23-1.53) | 0.22 | (0.08-0.5) |
| Chad | TCD | 1039 | 2.7 | 0.45 | (0.16-1.04) | 0.14 | (0.07-0.26) | 0.05 | (0.02-0.08) | 2.11 | (0.65-5.21) | 0.64 | (0.27-1.26) | 0.21 | (0.09-0.4) |
| China | CHN | 8154 | 1.7 | 1.33 | (0.35-3.61) | 0.41 | (0.15-0.89) | 0.13 | (0.06-0.26) | 5.93 | (1.68-15.09) | 1.8 | (0.74-3.7) | 0.57 | (0.27-1.08) |
| Colombia | COL | 6895 | 2 | 1.14 | (0.29-3.05) | 0.34 | (0.13-0.75) | 0.11 | (0.05-0.22) | 5.08 | (1.46-13.15) | 1.53 | (0.63-3.11) | 0.48 | (0.23-0.9) |
| Comoros | COM | 847 | 1.9 | 0.59 | (0.2-1.35) | 0.18 | (0.09-0.34) | 0.06 | (0.03-0.1) | 2.74 | (0.83-6.78) | 0.85 | (0.36-1.73) | 0.27 | (0.12-0.53) |
| Congo | COG | 2329 | 2.9 | 0.61 | (0.18-1.52) | 0.19 | (0.08-0.38) | 0.06 | (0.03-0.11) | 2.76 | (0.84-6.92) | 0.83 | (0.37-1.65) | 0.27 | (0.13-0.48) |
| Costa Rica | CRI | 10916 | 1.4 | 1.61 | (0.42-4.29) | 0.49 | (0.18-1.08) | 0.16 | (0.07-0.32) | 7.14 | (2.03-18.26) | 2.18 | (0.91-4.49) | 0.7 | (0.33-1.3) |
| Cote d'Ivoire | CIV | 1362 | 2.4 | 0.57 | (0.19-1.35) | 0.17 | (0.08-0.32) | 0.06 | (0.03-0.1) | 2.59 | (0.82-6.34) | 0.79 | (0.34-1.56) | 0.26 | (0.12-0.47) |
| Democratic Republic of the Congo | COD | 478 | 1.6 | 0.54 | (0.19-1.25) | 0.17 | (0.08-0.31) | 0.05 | (0.03-0.1) | 2.56 | (0.71-6.52) | 0.79 | (0.29-1.75) | 0.26 | (0.1-0.56) |
| Djibouti | DJI | 1805 | 1.8 | 0.89 | (0.27-2.21) | 0.27 | (0.12-0.53) | 0.09 | (0.04-0.16) | 4.06 | (1.27-9.9) | 1.23 | (0.55-2.4) | 0.4 | (0.19-0.74) |
| Dominica | DMA | 7554 | 1.5 | 1.52 | (0.4-4.08) | 0.46 | (0.17-1.04) | 0.15 | (0.06-0.3) | 6.8 | (1.89-17.2) | 2.06 | (0.85-4.2) | 0.66 | (0.31-1.24) |
| Dominican Republic | DOM | 6134 | 2.2 | 1.03 | (0.27-2.81) | 0.31 | (0.11-0.68) | 0.1 | (0.04-0.2) | 4.54 | (1.29-11.82) | 1.38 | (0.58-2.82) | 0.44 | (0.21-0.83) |
| Ecuador | ECU | 6307 | 1.8 | 1.26 | (0.32-3.44) | 0.38 | (0.14-0.84) | 0.12 | (0.05-0.24) | 5.54 | (1.56-14.2) | 1.69 | (0.71-3.45) | 0.54 | (0.25-1.03) |
| El Salvador | SLV | 4101 | 2 | 1.13 | (0.29-2.94) | 0.34 | (0.13-0.75) | 0.11 | (0.05-0.22) | 4.98 | (1.42-12.7) | 1.51 | (0.64-3.11) | 0.48 | (0.22-0.9) |
| Eritrea | ERI | 630 | 1.9 | 0.52 | (0.18-1.22) | 0.16 | (0.08-0.29) | 0.05 | (0.03-0.09) | 2.42 | (0.71-6.01) | 0.75 | (0.29-1.58) | 0.24 | (0.1-0.5) |
| Ethiopia | ETH | 619 | 2.8 | 0.35 | (0.12-0.8) | 0.11 | (0.05-0.19) | 0.03 | (0.02-0.06) | 1.62 | (0.48-4.08) | 0.5 | (0.2-1.06) | 0.16 | (0.07-0.33) |
| Fiji | FJI | 4959 | 1.7 | 1.33 | (0.35-3.56) | 0.4 | (0.15-0.87) | 0.13 | (0.05-0.26) | 5.82 | (1.63-14.8) | 1.77 | (0.73-3.66) | 0.57 | (0.26-1.06) |
| Gabon | GAB | 9343 | 2.5 | 0.89 | (0.23-2.41) | 0.27 | (0.1-0.59) | 0.09 | (0.04-0.18) | 3.9 | (1.1-9.99) | 1.19 | (0.5-2.45) | 0.38 | (0.18-0.72) |
| Gambia | GMB | 395 | 4.2 | 0.19 | (0.06-0.44) | 0.06 | (0.03-0.11) | 0.02 | (0.01-0.03) | 0.88 | (0.24-2.33) | 0.27 | (0.1-0.62) | 0.09 | (0.03-0.2) |
| Georgia | GEO | 3087 | 2.6 | 0.81 | (0.22-2.11) | 0.24 | (0.1-0.52) | 0.08 | (0.03-0.15) | 3.57 | (1.07-8.98) | 1.09 | (0.46-2.19) | 0.35 | (0.17-0.65) |
| Ghana | GHA | 1459 | 2.9 | 0.5 | (0.16-1.19) | 0.15 | (0.07-0.28) | 0.05 | (0.03-0.08) | 2.24 | (0.69-5.42) | 0.68 | (0.31-1.34) | 0.22 | (0.11-0.41) |
| Grenada | GRD | 8294 | 1.5 | 1.54 | (0.41-4.11) | 0.47 | (0.17-1.01) | 0.15 | (0.06-0.31) | 6.77 | (1.89-17.41) | 2.05 | (0.83-4.2) | 0.66 | (0.31-1.23) |
| Guatemala | GTM | 4060 | 1.9 | 1.2 | (0.32-3.18) | 0.36 | (0.14-0.8) | 0.12 | (0.05-0.24) | 5.38 | (1.54-13.92) | 1.61 | (0.67-3.3) | 0.52 | (0.25-0.98) |
| Guinea | GIN | 612 | 2.1 | 0.45 | (0.16-1.01) | 0.14 | (0.07-0.25) | 0.05 | (0.02-0.08) | 2.1 | (0.6-5.39) | 0.65 | (0.25-1.41) | 0.21 | (0.09-0.44) |
| Guinea-Bissau | GNB | 553 | 2.7 | 0.34 | (0.12-0.77) | 0.1 | (0.05-0.19) | 0.03 | (0.02-0.06) | 1.59 | (0.45-4.05) | 0.49 | (0.19-1.07) | 0.16 | (0.06-0.33) |
| Guyana | GUY | 3924 | 1.8 | 1.24 | (0.32-3.3) | 0.37 | (0.14-0.82) | 0.12 | (0.05-0.24) | 5.42 | (1.56-13.6) | 1.68 | (0.69-3.47) | 0.53 | (0.25-0.99) |
| Haiti | HTI | 863 | 2.1 | 0.53 | (0.18-1.23) | 0.16 | (0.08-0.3) | 0.05 | (0.03-0.09) | 2.47 | (0.75-6.02) | 0.76 | (0.32-1.55) | 0.25 | (0.11-0.48) |
| Honduras | HND | 2369 | 2 | 0.88 | (0.26-2.25) | 0.27 | (0.11-0.54) | 0.09 | (0.04-0.16) | 3.93 | (1.21-9.61) | 1.21 | (0.53-2.37) | 0.39 | (0.19-0.71) |
| Hungary | HUN | 12853 | 2 | 1.14 | (0.29-3.1) | 0.34 | (0.13-0.74) | 0.11 | (0.05-0.22) | 5.02 | (1.4-12.94) | 1.51 | (0.62-3.08) | 0.48 | (0.23-0.91) |
| India | IND | 1808 | 3.5 | 0.46 | (0.14-1.13) | 0.14 | (0.06-0.27) | 0.05 | (0.02-0.08) | 2.07 | (0.63-5) | 0.63 | (0.28-1.23) | 0.2 | (0.1-0.37) |
| Indonesia | IDN | 3511 | 3.2 | 0.7 | (0.19-1.86) | 0.21 | (0.08-0.46) | 0.07 | (0.03-0.13) | 3.09 | (0.89-7.84) | 0.93 | (0.39-1.87) | 0.3 | (0.14-0.56) |
| Iran (Islamic Republic of) | IRN | 4983 | 3.4 | 0.66 | (0.17-1.82) | 0.2 | (0.07-0.44) | 0.06 | (0.03-0.13) | 2.91 | (0.83-7.45) | 0.89 | (0.37-1.79) | 0.28 | (0.13-0.53) |
| Iraq | IRQ | 4701 | 3.1 | 0.74 | (0.19-1.97) | 0.22 | (0.08-0.5) | 0.07 | (0.03-0.15) | 3.26 | (0.95-8.28) | 0.99 | (0.41-1.99) | 0.32 | (0.15-0.6) |
| Jamaica | JAM | 4922 | 1.8 | 1.28 | (0.34-3.41) | 0.39 | (0.15-0.85) | 0.12 | (0.05-0.24) | 5.58 | (1.6-14.46) | 1.72 | (0.7-3.56) | 0.55 | (0.26-1.04) |
| Jordan | JOR | 5590 | 2.2 | 1.03 | (0.27-2.76) | 0.32 | (0.12-0.69) | 0.1 | (0.04-0.2) | 4.6 | (1.33-11.61) | 1.39 | (0.58-2.85) | 0.45 | (0.21-0.85) |
| Kazakhstan | KAZ | 11488 | 2.1 | 1.08 | (0.28-2.91) | 0.33 | (0.12-0.72) | 0.1 | (0.04-0.21) | 4.67 | (1.36-11.79) | 1.44 | (0.59-2.94) | 0.46 | (0.21-0.87) |
| Kenya | KEN | 1495 | 2.2 | 0.67 | (0.22-1.61) | 0.2 | (0.1-0.38) | 0.07 | (0.03-0.11) | 3 | (0.93-7.35) | 0.92 | (0.41-1.82) | 0.3 | (0.14-0.55) |
| Kiribati | KIR | 1511 | 1.2 | 1.25 | (0.4-3) | 0.38 | (0.18-0.72) | 0.12 | (0.06-0.21) | 5.63 | (1.75-13.96) | 1.73 | (0.76-3.4) | 0.56 | (0.26-1.04) |
| Kyrgyzstan | KGZ | 1280 | 2.7 | 0.5 | (0.16-1.2) | 0.15 | (0.07-0.28) | 0.05 | (0.03-0.09) | 2.28 | (0.7-5.51) | 0.7 | (0.3-1.38) | 0.23 | (0.11-0.43) |
| Lao People's Democratic Republic | LAO | 1816 | 2.9 | 0.54 | (0.17-1.34) | 0.17 | (0.08-0.32) | 0.05 | (0.03-0.09) | 2.43 | (0.76-6.03) | 0.74 | (0.33-1.46) | 0.24 | (0.12-0.45) |
| Lebanon | LBN | 12006 | 1.5 | 1.48 | (0.39-3.96) | 0.45 | (0.17-0.99) | 0.14 | (0.06-0.29) | 6.5 | (1.81-16.65) | 1.98 | (0.81-4.06) | 0.64 | (0.3-1.2) |
| Lesotho | LSO | 1103 | 2.6 | 0.48 | (0.16-1.13) | 0.15 | (0.07-0.27) | 0.05 | (0.03-0.08) | 2.16 | (0.68-5.29) | 0.67 | (0.29-1.34) | 0.22 | (0.1-0.41) |
| Liberia | LBR | 479 | 1.8 | 0.48 | (0.17-1.09) | 0.15 | (0.07-0.27) | 0.05 | (0.02-0.09) | 2.26 | (0.61-5.73) | 0.7 | (0.26-1.53) | 0.23 | (0.09-0.49) |
| Libya | LBY | 5430 | 3 | 0.76 | (0.2-2.03) | 0.23 | (0.09-0.51) | 0.07 | (0.03-0.15) | 3.34 | (0.93-8.55) | 1.01 | (0.42-2.07) | 0.32 | (0.15-0.6) |
| Madagascar | MDG | 444 | 3.3 | 0.25 | (0.08-0.57) | 0.08 | (0.04-0.14) | 0.02 | (0.01-0.04) | 1.17 | (0.32-3.05) | 0.36 | (0.13-0.8) | 0.12 | (0.04-0.26) |
| Malawi | MWI | 273 | 3 | 0.23 | (0.08-0.54) | 0.07 | (0.03-0.14) | 0.02 | (0.01-0.04) | 1.09 | (0.28-2.97) | 0.34 | (0.11-0.81) | 0.11 | (0.03-0.27) |
| Malaysia | MYS | 10654 | 2.4 | 0.95 | (0.25-2.56) | 0.29 | (0.11-0.63) | 0.09 | (0.04-0.18) | 4.16 | (1.23-10.5) | 1.27 | (0.52-2.61) | 0.4 | (0.19-0.75) |
| Maldives | MDV | 8717 | 1.7 | 1.32 | (0.34-3.61) | 0.4 | (0.15-0.89) | 0.13 | (0.05-0.26) | 5.82 | (1.68-14.78) | 1.78 | (0.74-3.69) | 0.57 | (0.27-1.06) |
| Mali | MLI | 673 | 2.7 | 0.37 | (0.13-0.83) | 0.12 | (0.06-0.21) | 0.04 | (0.02-0.06) | 1.74 | (0.51-4.25) | 0.54 | (0.21-1.13) | 0.17 | (0.07-0.35) |
| Marshall Islands | MHL | 3527 | 1 | 2.31 | (0.62-6.05) | 0.7 | (0.27-1.51) | 0.22 | (0.1-0.45) | 10.27 | (3-25.85) | 3.13 | (1.28-6.32) | 1 | (0.47-1.88) |
| Mauritania | MRT | 1347 | 3.3 | 0.41 | (0.14-0.97) | 0.13 | (0.06-0.24) | 0.04 | (0.02-0.07) | 1.89 | (0.59-4.56) | 0.58 | (0.26-1.14) | 0.19 | (0.09-0.35) |
| Mauritius | MUS | 10609 | 1.8 | 1.25 | (0.32-3.41) | 0.38 | (0.14-0.84) | 0.12 | (0.05-0.24) | 5.44 | (1.58-14.1) | 1.67 | (0.69-3.45) | 0.54 | (0.25-1.01) |
| Mexico | MEX | 10174 | 1.8 | 1.26 | (0.33-3.44) | 0.38 | (0.14-0.82) | 0.12 | (0.05-0.24) | 5.55 | (1.61-14.36) | 1.69 | (0.71-3.48) | 0.54 | (0.26-1.02) |
| Micronesia (Federated States of) | FSM | 3052 | 1 | 2.06 | (0.56-5.38) | 0.62 | (0.24-1.3) | 0.2 | (0.09-0.38) | 9.07 | (2.63-22.46) | 2.76 | (1.18-5.45) | 0.89 | (0.42-1.64) |
| Mongolia | MNG | 4195 | 2.9 | 0.77 | (0.2-2.03) | 0.24 | (0.09-0.52) | 0.08 | (0.03-0.15) | 3.4 | (0.97-8.55) | 1.04 | (0.43-2.14) | 0.33 | (0.16-0.63) |
| Montenegro | MNE | 6778 | 2.3 | 0.98 | (0.25-2.6) | 0.29 | (0.11-0.64) | 0.09 | (0.04-0.19) | 4.3 | (1.23-10.84) | 1.31 | (0.53-2.72) | 0.42 | (0.2-0.78) |
| Morocco | MAR | 3046 | 2.6 | 0.79 | (0.23-1.99) | 0.24 | (0.1-0.51) | 0.08 | (0.03-0.14) | 3.51 | (1.02-8.76) | 1.06 | (0.46-2.14) | 0.34 | (0.16-0.64) |
| Mozambique | MOZ | 624 | 2 | 0.49 | (0.17-1.13) | 0.15 | (0.07-0.27) | 0.05 | (0.03-0.09) | 2.28 | (0.66-5.65) | 0.7 | (0.28-1.48) | 0.23 | (0.09-0.48) |
| Myanmar | MMR | 1334 | 3.8 | 0.35 | (0.12-0.84) | 0.11 | (0.05-0.2) | 0.04 | (0.02-0.06) | 1.62 | (0.5-3.92) | 0.5 | (0.22-0.98) | 0.16 | (0.08-0.31) |
| Namibia | NAM | 5691 | 2 | 1.13 | (0.29-3.09) | 0.34 | (0.13-0.74) | 0.11 | (0.05-0.22) | 4.93 | (1.4-12.79) | 1.53 | (0.62-3.08) | 0.49 | (0.23-0.91) |
| Nepal | NPL | 763 | 3.3 | 0.32 | (0.11-0.74) | 0.1 | (0.05-0.18) | 0.03 | (0.02-0.05) | 1.48 | (0.46-3.68) | 0.46 | (0.19-0.96) | 0.15 | (0.06-0.3) |
| Nicaragua | NIC | 1952 | 2.5 | 0.66 | (0.19-1.62) | 0.2 | (0.09-0.39) | 0.06 | (0.03-0.11) | 2.91 | (0.9-7.01) | 0.89 | (0.4-1.76) | 0.29 | (0.14-0.53) |
| Niger | NER | 412 | 2.6 | 0.31 | (0.1-0.71) | 0.1 | (0.05-0.18) | 0.03 | (0.02-0.06) | 1.44 | (0.4-3.68) | 0.45 | (0.16-1.02) | 0.15 | (0.05-0.33) |
| Nigeria | NGA | 2884 | 2.2 | 0.93 | (0.26-2.44) | 0.28 | (0.11-0.58) | 0.09 | (0.04-0.17) | 4.12 | (1.22-10.22) | 1.25 | (0.54-2.49) | 0.4 | (0.19-0.74) |
| Palau | PLW | 15531 | 1.1 | 2.1 | (0.55-5.58) | 0.65 | (0.24-1.41) | 0.21 | (0.09-0.41) | 9.27 | (2.74-23.1) | 2.85 | (1.18-5.82) | 0.91 | (0.42-1.72) |
| Panama | PAN | 11851 | 1.7 | 1.33 | (0.34-3.53) | 0.4 | (0.15-0.87) | 0.13 | (0.05-0.26) | 5.84 | (1.67-14.96) | 1.78 | (0.73-3.72) | 0.56 | (0.27-1.05) |
| Papua New Guinea | PNG | 2597 | 1.1 | 1.74 | (0.49-4.46) | 0.53 | (0.22-1.08) | 0.17 | (0.08-0.32) | 7.82 | (2.3-19.35) | 2.36 | (1.04-4.69) | 0.76 | (0.37-1.39) |
| Paraguay | PRY | 4383 | 2 | 1.15 | (0.3-3.08) | 0.34 | (0.13-0.74) | 0.11 | (0.05-0.22) | 5.05 | (1.42-12.99) | 1.54 | (0.63-3.12) | 0.49 | (0.23-0.93) |
| Peru | PER | 5962 | 2 | 1.12 | (0.29-3.03) | 0.34 | (0.13-0.74) | 0.11 | (0.05-0.22) | 4.97 | (1.46-12.86) | 1.49 | (0.61-3.1) | 0.47 | (0.23-0.89) |
| Philippines | PHL | 3037 | 2.4 | 0.84 | (0.24-2.19) | 0.26 | (0.1-0.54) | 0.08 | (0.04-0.16) | 3.74 | (1.11-9.37) | 1.14 | (0.48-2.28) | 0.37 | (0.18-0.68) |
| Republic of Moldova | MDA | 1726 | 2.9 | 0.54 | (0.17-1.29) | 0.16 | (0.08-0.32) | 0.05 | (0.03-0.09) | 2.42 | (0.77-5.86) | 0.75 | (0.33-1.47) | 0.24 | (0.12-0.43) |
| Romania | ROU | 9570 | 2.1 | 1.06 | (0.27-2.78) | 0.32 | (0.12-0.72) | 0.1 | (0.04-0.21) | 4.69 | (1.32-12.12) | 1.42 | (0.58-2.92) | 0.46 | (0.21-0.85) |
| Rwanda | RWA | 769 | 2.3 | 0.45 | (0.16-1.03) | 0.14 | (0.07-0.25) | 0.05 | (0.02-0.08) | 2.11 | (0.62-5.3) | 0.64 | (0.27-1.35) | 0.21 | (0.09-0.42) |
| Saint Lucia | LCA | 8250 | 1.4 | 1.61 | (0.42-4.41) | 0.48 | (0.18-1.05) | 0.15 | (0.06-0.31) | 7.03 | (2.05-18.04) | 2.14 | (0.87-4.32) | 0.68 | (0.33-1.28) |
| Saint Vincent and the Grenadines | VCT | 6894 | 1.6 | 1.41 | (0.37-3.73) | 0.43 | (0.16-0.94) | 0.14 | (0.06-0.28) | 6.25 | (1.84-16.18) | 1.89 | (0.78-3.8) | 0.61 | (0.28-1.15) |
| Samoa | WSM | 4487 | 1.2 | 1.92 | (0.49-5.15) | 0.58 | (0.22-1.27) | 0.19 | (0.08-0.37) | 8.43 | (2.51-21.05) | 2.57 | (1.06-5.34) | 0.83 | (0.39-1.55) |
| Sao Tome and Principe | STP | 1912 | 1.7 | 0.95 | (0.3-2.31) | 0.29 | (0.13-0.56) | 0.09 | (0.05-0.17) | 4.28 | (1.29-10.68) | 1.31 | (0.58-2.57) | 0.42 | (0.2-0.78) |
| Senegal | SEN | 1006 | 2.4 | 0.51 | (0.17-1.19) | 0.16 | (0.08-0.28) | 0.05 | (0.03-0.08) | 2.33 | (0.71-5.74) | 0.72 | (0.31-1.45) | 0.23 | (0.11-0.45) |
| Serbia | SRB | 5676 | 2.4 | 0.97 | (0.25-2.62) | 0.29 | (0.11-0.64) | 0.09 | (0.04-0.19) | 4.26 | (1.21-11.01) | 1.29 | (0.53-2.61) | 0.42 | (0.2-0.78) |
| Seychelles | SYC | 14500 | 1.8 | 1.24 | (0.32-3.3) | 0.38 | (0.14-0.83) | 0.12 | (0.05-0.24) | 5.49 | (1.57-14.26) | 1.67 | (0.69-3.46) | 0.54 | (0.25-1.01) |
| Sierra Leone | SLE | 696 | 2.5 | 0.4 | (0.14-0.91) | 0.12 | (0.06-0.23) | 0.04 | (0.02-0.07) | 1.85 | (0.55-4.57) | 0.57 | (0.23-1.17) | 0.19 | (0.08-0.37) |
| Solomon Islands | SLB | 2052 | 0.9 | 1.78 | (0.53-4.35) | 0.54 | (0.24-1.05) | 0.18 | (0.09-0.32) | 8.05 | (2.49-19.71) | 2.43 | (1.08-4.73) | 0.79 | (0.39-1.45) |
| South Africa | ZAF | 5902 | 2.2 | 1.03 | (0.26-2.76) | 0.31 | (0.11-0.68) | 0.1 | (0.04-0.2) | 4.44 | (1.3-11.29) | 1.35 | (0.57-2.8) | 0.44 | (0.21-0.81) |
| South Sudan | SSD | 1029 | 2.2 | 0.54 | (0.18-1.24) | 0.17 | (0.08-0.31) | 0.05 | (0.03-0.09) | 2.53 | (0.78-6.16) | 0.76 | (0.33-1.51) | 0.25 | (0.11-0.48) |
| Sri Lanka | LKA | 3818 | 2.9 | 0.78 | (0.2-2.09) | 0.24 | (0.09-0.52) | 0.08 | (0.03-0.15) | 3.48 | (0.99-8.8) | 1.05 | (0.44-2.14) | 0.34 | (0.16-0.64) |
| Sudan | SDN | 1983 | 2.2 | 0.76 | (0.23-1.87) | 0.23 | (0.1-0.46) | 0.07 | (0.04-0.13) | 3.38 | (1.05-8.16) | 1.05 | (0.46-2.07) | 0.34 | (0.17-0.61) |
| Suriname | SUR | 9950 | 1.7 | 1.32 | (0.34-3.6) | 0.4 | (0.15-0.87) | 0.13 | (0.05-0.25) | 5.85 | (1.7-14.87) | 1.78 | (0.74-3.6) | 0.57 | (0.27-1.06) |
| Swaziland | SWZ | 3248 | 2.4 | 0.87 | (0.24-2.29) | 0.27 | (0.1-0.57) | 0.08 | (0.04-0.16) | 3.83 | (1.13-9.65) | 1.18 | (0.49-2.35) | 0.38 | (0.18-0.69) |
| Tajikistan | TJK | 1007 | 2.7 | 0.44 | (0.15-1.03) | 0.14 | (0.07-0.25) | 0.04 | (0.02-0.07) | 2.03 | (0.63-5.03) | 0.62 | (0.27-1.23) | 0.2 | (0.09-0.38) |
| Thailand | THA | 5612 | 2.7 | 0.86 | (0.22-2.32) | 0.26 | (0.1-0.57) | 0.08 | (0.03-0.17) | 3.74 | (1.08-9.44) | 1.15 | (0.48-2.34) | 0.37 | (0.17-0.69) |
| The Former Yugoslav Republic of Macedonia | MKD | 4896 | 2.9 | 0.79 | (0.21-2.15) | 0.24 | (0.09-0.53) | 0.08 | (0.03-0.16) | 3.5 | (1.01-8.87) | 1.07 | (0.44-2.19) | 0.34 | (0.16-0.65) |
| Timor-Leste | TLS | 3114 | 1.7 | 1.2 | (0.33-3.02) | 0.37 | (0.15-0.79) | 0.12 | (0.05-0.23) | 5.37 | (1.57-13.33) | 1.64 | (0.7-3.28) | 0.53 | (0.25-1) |
| Togo | TGO | 628 | 2.4 | 0.4 | (0.14-0.91) | 0.12 | (0.06-0.22) | 0.04 | (0.02-0.07) | 1.87 | (0.56-4.77) | 0.58 | (0.23-1.21) | 0.19 | (0.08-0.39) |
| Tonga | TON | 4639 | 1.1 | 2.1 | (0.55-5.7) | 0.63 | (0.24-1.39) | 0.2 | (0.09-0.41) | 9.22 | (2.64-23.81) | 2.82 | (1.17-5.71) | 0.9 | (0.42-1.68) |
| Tunisia | TUN | 4075 | 2.9 | 0.8 | (0.2-2.12) | 0.24 | (0.09-0.52) | 0.08 | (0.03-0.16) | 3.51 | (1.01-8.87) | 1.07 | (0.44-2.2) | 0.34 | (0.16-0.63) |
| Turkey | TUR | 9680 | 2.1 | 1.09 | (0.28-2.94) | 0.33 | (0.12-0.74) | 0.11 | (0.04-0.22) | 4.82 | (1.32-12.23) | 1.44 | (0.6-2.96) | 0.47 | (0.22-0.88) |
| Turkmenistan | TKM | 7585 | 2 | 1.12 | (0.29-2.98) | 0.34 | (0.13-0.74) | 0.11 | (0.05-0.22) | 4.96 | (1.37-12.62) | 1.51 | (0.61-3.13) | 0.48 | (0.23-0.9) |
| Tuvalu | TUV | 3258 | 1 | 2.02 | (0.55-5.35) | 0.61 | (0.24-1.35) | 0.2 | (0.09-0.39) | 8.96 | (2.66-22.51) | 2.71 | (1.13-5.4) | 0.88 | (0.41-1.63) |
| Uganda | UGA | 684 | 3.1 | 0.33 | (0.11-0.75) | 0.1 | (0.05-0.18) | 0.03 | (0.02-0.06) | 1.52 | (0.45-3.93) | 0.47 | (0.19-1) | 0.15 | (0.06-0.31) |
| Ukraine | UKR | 2002 | 4.1 | 0.4 | (0.12-1) | 0.12 | (0.06-0.24) | 0.04 | (0.02-0.07) | 1.8 | (0.55-4.44) | 0.55 | (0.24-1.09) | 0.18 | (0.09-0.33) |
| United Republic of Tanzania | TZA | 1018 | 2.8 | 0.44 | (0.15-1) | 0.13 | (0.07-0.24) | 0.04 | (0.02-0.07) | 1.99 | (0.63-4.86) | 0.62 | (0.26-1.22) | 0.2 | (0.09-0.38) |
| Uzbekistan | UZB | 2202 | 2.7 | 0.64 | (0.19-1.62) | 0.2 | (0.09-0.39) | 0.06 | (0.03-0.12) | 2.89 | (0.89-7.13) | 0.88 | (0.39-1.74) | 0.28 | (0.14-0.51) |
| Vanuatu | VUT | 2775 | 0.9 | 16.06 | (2.24-60.07) | 4.86 | (0.83-16.29) | 1.53 | (0.29-5) | 70.6 | (10.41-252.97) | 21.3 | (4.14-66.85) | 6.73 | (1.4-20.08) |
| Venezuela (Bolivarian Republic of) | VEN | 5485 | 3 | 0.77 | (0.2-2.06) | 0.23 | (0.09-0.5) | 0.07 | (0.03-0.15) | 3.35 | (0.97-8.42) | 1.02 | (0.43-2.09) | 0.33 | (0.16-0.61) |
| Viet Nam | VNM | 2233 | 2.7 | 0.66 | (0.2-1.64) | 0.2 | (0.09-0.4) | 0.06 | (0.03-0.12) | 2.94 | (0.89-7.11) | 0.89 | (0.39-1.73) | 0.29 | (0.14-0.52) |
| Yemen | YEM | 1525 | 2.4 | 0.61 | (0.19-1.46) | 0.19 | (0.09-0.36) | 0.06 | (0.03-0.11) | 2.78 | (0.85-6.8) | 0.85 | (0.38-1.67) | 0.27 | (0.13-0.51) |
| Zambia | ZMB | 1833 | 2.3 | 0.69 | (0.21-1.72) | 0.21 | (0.09-0.41) | 0.07 | (0.03-0.12) | 3.11 | (0.96-7.7) | 0.95 | (0.42-1.87) | 0.3 | (0.15-0.55) |
| Zimbabwe | ZWE | 1042 | 2 | 0.6 | (0.2-1.4) | 0.18 | (0.09-0.34) | 0.06 | (0.03-0.1) | 2.77 | (0.84-6.63) | 0.85 | (0.36-1.7) | 0.28 | (0.13-0.53) |

**^1^ Gross Domestic Product (GDP) per capita (2015 US$)**

**^2^ Purchasing Power Parity (PPP) conversion factor (international dollars per US$)**
